# Wheat Genomics: Present Status and Future Prospects

**DOI:** 10.1155/2008/896451

**Published:** 2008-05-19

**Authors:** P. K. Gupta, R. R. Mir, A. Mohan, J. Kumar

**Affiliations:** Molecular Biology Laboratory, Department of Genetics and Plant Breeding, Ch. Charan Singh University, Meerut 250 004, India

## Abstract

Wheat (*Triticum aestivum* L.), with a large genome (16000 Mb) and high proportion (∼80%) of repetitive sequences, has been a difficult crop for genomics research. However, the availability of extensive cytogenetics stocks has been an asset, which facilitated significant progress in wheat genomic research in recent years. For instance, fairly dense molecular maps (both genetic and physical maps) and a large set of ESTs allowed genome-wide identification of gene-rich and gene-poor regions as well as QTL including eQTL. The availability of markers associated with major economic traits also allowed development of major programs on marker-assisted selection (MAS) in some countries, and facilitated map-based cloning of a number of genes/QTL. Resources for functional genomics including TILLING and RNA interference (RNAi) along with some new approaches like epigenetics and association mapping are also being successfully used for wheat genomics research. BAC/BIBAC libraries for the subgenome D and some individual chromosomes have also been prepared to facilitate sequencing of gene space. In this brief review, we discuss all these advances in some detail, and also describe briefly the available resources, which can be used for future genomics research in this important crop.

## 1. INTRODUCTION

Wheat is one of the most important staple food crops of the world, occupying 17% (one sixth) of crop acreage worldwide, feeding about 40% (nearly half) of the world population and providing 20% (one fifth) of total food calories and protein in human nutrition. Although wheat production during the last four decades had a steady significant increase, a fatigue has been witnessed during the last few years, leading to the lowest current global wheat stocks ever since 1948/49. Consequently, wheat prices have also been soaring, reaching the highest level of US $ 10 a bushel as against US $ 4.50 a year ago (http://www.planetark.com/dailynewsstory.cfm/newsid/ 44968/story.htm). As against this, it is projected that, in order to meet growing human needs, wheat grain production must increase at an annual rate of 2%, without any additional land to become available for this crop [1]. In order to meet this challenge, new level of understanding of the structure and function of the wheat genome is required.

Wheat is adapted to temperate regions of the world and was one of the first crops to be domesticated some 10000 years ago. At the cytogenetics level, common wheat is known to have three subgenomes (each subgenome has 7 chromosomes, making *n* = 21) that are organized in seven homoeologous groups, each homoeologous group has three closely related chromosomes, one from each of the three related subgenomes. The diploid progenitors of the A, B, and D subgenomes have been identified, although there has always been a debate regarding the progenitor of B genome (reviewed in [1]). It has also been found that common wheat behaves much like a diploid organism during meiosis, but its genome can tolerate aneuploidy because of the presence of triplicate genes. These features along with the availability of a large number of aneuploids [particularly including a complete set of monosomics, a set of 42 compensating nullisomic-tetrasomics and a complete set of 42 ditelocentrics developed by Sears [2]] and more than 400 segmental deletion lines [developed later by Endo and Gill [3]] facilitated greatly the wheat genomics research.

Molecular tools have recently been used in a big way for cytogenetic studies in wheat, so that all recent cytogenetic studies in wheat now have a molecular component, thus paving the path for wheat genomics research. However, these studies in the area of molecular cytogenetics have been relatively difficult in bread wheat due to its three closely related subgenomes and a large genome (1C = >16 billion base pairs) with high proportion (>80%) of repetitive DNA. Despite this, significant progress in the area of molecular cytogenetics and cytogenomics of wheat has been made during the last two decades, thus making it amenable to genomics research. For instance, molecular maps in bread wheat, emmer wheat, and einkorn wheat utilizing a variety of molecular markers are now available, where gene rich regions (GRRs) and recombination hotspots have also been identified (for a review, see [4, 5]).

In recent years, a number of initiatives have been taken to develop new tools for wheat genomics research. These include construction of large insert libraries and development of massive EST collections, genetic and physical molecular maps, and gene targeting systems. For instance, the number of wheat ESTs has increased from a mere ∼5 in 1999 [6] to a massive >1 240 000 in January 2008 (http://www.ncbi.nlm.nih.gov/), thus forming the largest EST collection in any crop as a resource for genome analysis. These ESTs are being used for a variety of activities including development of functional molecular markers, preparation of transcript maps, and construction of cDNA arrays. A variety of molecular markers that were developed either from ESTs or from genomic DNA also helped to discover relationships between genomes [7] and to compare marker-trait associations in different crops. Comparative genomics, involving major crop grasses including wheat, has also been used not only to study evolutionary relationships, but also to design crop improvement programs [8]. Functional genomics research in wheat, which though lagged far behind relative to that in other major food crops like maize and rice, has also recently witnessed significant progress. For instance, RNA interference, TILLING, and “expression genetics” leading to mapping of eQTLs have been used to identify functions of individual genes [9]. This allowed development of sets of candidate genes for individual traits, which can be used for understanding the biology of these traits and for development of perfect diagnostic marker(s) to be used not only for map-based cloning of genes, but also for MAS [9, 10]. In order to sequence the GRRs of wheat genome, a multinational collaborative program named International Genome Research on Wheat (IGROW) was earlier launched, which later took the shape of International Wheat Genome Sequencing Consortium (IWGSC) [11]. This will accelerate the progress on genome sequencing and will allow analysis of structure and function of the wheat genome. Keeping the above background in mind, Somers [12] identified the following five thrust areas of research for wheat improvement: (i) genetic mapping, (ii) QTL analysis, (iii) molecular breeding, (iv) association mapping, and (v) software development. In this communication, we briefly review the recent advances in all these areas of wheat genomics and discuss their impact on wheat improvement programs.

## 2. MOLECULAR MAPS OF WHEAT GENOME

### 2.1. Molecular genetic maps

Although some efforts toward mapping of molecular markers on wheat genome were initially made during late 1980s [13], a systematic construction of molecular maps in wheat started only in 1990, with the organization of International Triticeae Mapping Initiative (ITMI), which coordinated the construction of molecular maps of wheat genome. Individual groups (headed by R Appels, PJ Sharp, ME Sorrells, J Dvorak, BS Gill, GE Hart, and MD Gale) prepared the maps for chromosomes belonging to each of the seven different homoeologous groups. A detailed account on mapping of chromosomes of individual homoeologous groups and that of the whole wheat genome is available elsewhere [14]; an updated version is available at GrainGenes (http://wheat.pw.usda.gov/), and summarized in Table 1. Integrated or composite maps involving more than one type of molecular markers have also been prepared in wheat (particularly the SSR, AFLP, SNP, and DArT markers (see Table 1)). Consensus maps, where map information from multiple genomes or multiple maps was merged into a single comprehensive map, were also prepared in wheat [15, 16]. On these maps, classical and newly identified genes of economic importance are being placed to facilitate marker-assisted selection (MAS). Many genes controlling a variety of traits (both qualitative and quantitative) have already been tagged/mapped using a variety of molecular markers (for references, see [14, 17]). The density of wheat genetic maps was improved with the development of microsatellite (SSR) markers leading to construction of SSR maps of wheat [18–20]. Later, Somers et al. [16] added more SSR markers to these earlier maps and prepared a high-density SSR consensus map. At present, >2500 mapped genomic SSR (gSSR) markers are available in wheat, which will greatly facilitate the preparation of high-density genetic maps, so that we will be able to identify key recombination events in breeding populations and fine-map genes. In addition to gSSRs, more then 300 EST-SSR could also be placed on the genetic map of wheat genome [21–23]. However, more markers are still needed, particularly for preparation of high-density physical maps for gene cloning [24]. Availability of a number of molecular markers associated each with individual traits will also facilitate marker-assisted selection (MAS) during plant breeding.

In addition to random DNA markers (RDM), gene targeted markers (GTMs) and functional markers (FMs) are also being used in wheat to facilitate identification of genes responsible for individual traits and to improve possibilities of using MAS in wheat breeding. As a corollary, functional markers (FMs) are also being developed from the available gene sequences [10]. These markers were also used to construct transcript and molecular functional maps. Recently, microarray-based high-throughput diversity array technology (DArT) markers were also developed and used for preparing genetic maps in wheat [53, 54]. Large-scale genotyping for dozens to thousands of SNPs is also being undertaken using several high-density platforms including Illumina’s GoldenGate and ABI’s SNaPshot platforms (http://wheat.pw.usda.gov/SNP/new/index.shtml). The genotyping activity may be extended further through the use of Solexa’s high throughput and low-cost resequencing technology.

### 2.2. Molecular marker-based physical maps

Molecular markers in bread wheat have also been used for the preparation of physical maps, which were then compared with the available genetic maps involving same markers. These maps allowed comparisons between genetic and physical distances to give information about variations in recombination frequencies and cryptic structural changes (if any) in different regions of individual chromosomes. Several methods have been employed for the construction of physical maps.

#### 2.2.1. Deletion mapping

In wheat, physical mapping of genes to individual chromosomes began with the development of aneuploids [55], which led to mapping of genes to individual chromosomes. Later, deletion lines of wheat chromosomes developed by Endo and Gill [3] were extensively used as a tool for physical mapping of molecular markers. Using these deletion stocks, genes for morphological characters were also mapped to physical segments of wheat chromosomes directly in case of unique and genome specific markers or indirectly in case of duplicate or triplicate loci through the use of intergenomic polymorphism between the A, B, and D subgenomes (see Table 2 for details of available physical maps). In addition to physical mapping of genomic SSRs, ESTs and EST-SSRs were also subjected to physical mapping (see Table 2). As a part of this effort, a major project (funded by National Science Foundation, USA) on mapping of ESTs in wheat was successfully completed by a consortium of 13 laboratories in USA leading to physical mapping of ∼16000 EST loci (http://wheat.pw.usda.gov/NSF/progress_mapping.html; [56] (see Table 2)).

#### 2.2.2. In silico physical mapping

As many as 16000 wheat EST loci assigned to deletion bins, as mentioned above, constitute a useful source for in silico mapping, so that markers with known sequences can be mapped to wheat chromosomes through sequence similarity with mapped EST loci available at GrainGene database (http://wheat.pw.usda.gov/GG2/blast.shtml). Using the above approach, Parida et al. [80] were able to map 157 SSR containing wheat unique sequences (out of 429 class I unigene-derived microsatellites (UGMS) markers developed in wheat) to chromosome bins. These bin-mapped UGMS markers provide valuable information for a targeted mapping of genes for useful traits, for comparative genomics, and for sequencing of gene-rich regions of the wheat genome. Another set of 672 loci belonging to 275 EST-SSRs of wheat and rye was assigned to individual bins through in silico and wet-lab approaches by Mohammedan et al. [79]. A few cDNA clones associated with QTL for FHB resistance in wheat were also successfully mapped using in silico approach [81].

#### 2.2.3. Radiation-hybrid mapping

Radiation hybrid (RH) mapping was first described by Goss and Harris [82] and was initially used by Cox et al. [83] for physical mapping in animals/humans. In wheat, the approach has been used at North Dakota State University (NDSU) utilizaing addition and substituition of individual D-genome chromosomes into tetraploid durum wheat. For RH mapping of 1D, durum wheat alien substitution line for chromosome 1D (DWRH-1D), harboring nuclear-cytoplasmic compatibility gene *scs^ae^* was used. These RH lines initially allowed detection of 88 radiation-induced breaks involving 39 1D specific markers. Later, this 1D RH map was further expanded to a resolution of one break every 199 kb of DNA, utilizing 378 markers [84]. Using the same approach, construction of radiation hybrid map for chromosome 3B is currently in progress (S. Kianian personal communication).

### 2.3. BAC-based physical maps

BAC-based physical map of wheat D genome is being constructed using the diploid species, *Aegilops tauschii*, with the aim to identify and map genes and later sequence the gene-rich regions (GRRs). For this purpose, a large number of BACs were first fingerprinted and assembled into contigs. Fingerprint contigs (FPCs) and the data related to physical mapping of the D genome are available in the database (http://wheat.pw.usda.gov/PhysicalMapping/index.html). BACs belonging to chromosome 3B are also being fingerprinted (with few BACs already anchored to wheat bins), and a whole genome BAC-based physical map of hexaploid wheat is proposed to be constructed under the aegis of IWGSC in its pilot studies (see later).

## 3. IN SITU HYBRIDIZATION STUDIES IN WHEAT

In bread wheat, in situ hybridization (ISH) involving radioactively labeled probes was initially used to localize repetitive DNA sequences, rRNA and alien DNA segments [104–106]. Later, fluorescence in situ hybridization (FISH), multicolor FISH (McFISH, simultaneous detection of more than one probe), and genome in situ hybridization (GISH, total genomic DNA as probe) were used in several studies. FISH with some repeated sequences as probes was used for identification of individual chromosomes [107–110]. FISH was also utilized to physically map rRNA multigene family [111, 112], RFLP markers [110, 113], and unique sequences [114–116] and also for detecting and locating alien chromatin introgressed into wheat [117–119].

A novel high-resolution FISH strategy using super-stretched flow-sorted chromosomes was also used (extended DNA fibre-FISH; [120–122]) to fine map DNA sequences [123, 124] and to confirm integration of transgenes into the wheat genome [125].

Recently, BACs were also utilized as probes for the so called BAC-FISH which helped not only in the discrimination between the three subgenomes, but also in the identification of intergenomic translocations, molecular cytogenetic markers, and individual chromosomes [126]. BAC-FISH also helped in localization of genes (BACs carrying genes) and in studying genome evolution and organization among wheat and its relatives [110, 127, 128].

## 4. MAP-BASED CLONING IN WHEAT

In wheat, a number of genes for some important traits including disease resistance, vernalization response, grain protein content, free threshing habit, and tolerance to abiotic stresses have been recently cloned/likely to be cloned via map-based cloning (see Table 3). The first genes to be isolated from wheat by map-based cloning included three resistance genes, against fungal diseases, including leaf rust (*Lr21*; [88, 129, 130] and *Lr10*; [87]) and powdery mildew (*Pm3b* ; [94]). A candidate gene for the *Q* locus conferring free threshing character to domesticated wheat was also cloned [92]. This gene influences many other domestication-related traits like glume shape and tenacity, rachis fragility, plant height, spike length, and ear-emergence time. Another important QTL, *Gpc-B1*, associated with increased grain protein, zinc, and iron content has been cloned, which will contribute in breeding enhanced nutritional value wheat in future [96]. Cloning of three genes for vernalization response (*VRN1, VRN2, VRN3*) helped in postulating a hypothetical model summarizing interactions among these three genes [89–91, 131].

## 5. EST DATABASES AND THEIR USES

During the last 8–10 years, more than 1240455 wheat ESTs have become available in the public domain as in January 2008 (http://www.ncbi.nlm.nih.gov/). A number of cDNA libraries have been used for this purpose. These ESTs proved to be an enormous resource for a variety of studies including development of functional molecular markers (particularly SSRs and SNPs), construction of a DNA chip, gene expression, genome organization, and comparative genomics research.

### 5.1. EST-derived SSRs

Wheat ESTs have been extensively used for SSR mining (1SSR/10.6 kb; [80]), so that in our own laboratory and elsewhere detected by author, a large number of SSRs have already been developed from EST sequences [22, 78, 80, 132–134]. These EST-SSRs served as a valuable source for a variety of studies including gene mapping, marker-aided selection (MAS), and eventually positional cloning of genes. The ESTs and EST-derived SSRs were also subjected to genetic and physical mapping (see above).

Since EST-SSRs are derived from the expressed portion of the genome, which is relatively more conserved, these markers show high level of transferability among species and genera [133, 135]. However, the transferability of wheat EST-SSRs to closely related triticeae species (*Triticum* and *Aegilops* species) is higher as compared to more distant relatives such as barley, maize, rice, sorghum, oats, and rye. The EST-SSRs thus also prove useful in comparative mapping, transfer of markers to orphanage wild species, and for genetic diversity estimates [79, 132, 134, 136–139].

### 5.2. EST-derived SNPs and the International SNP Consortium

In recent years, single nucleotide polymorphisms (SNPs) have become the markers of choice. Therefore, with the aim to discover and map SNPs in tetraploid and hexaploid wheats, an International Wheat SNP Consortium was constituted, and comprehensive wheat SNP database was developed (http://wheat.pw.usda.gov/SNP/new/index.shtml). Approximately 6000 EST unigenes from the database of mapped ESTs and other EST databases were distributed to consortium members for locating SNPs, for designing conserved primers for these SNPs and for validation of these SNP. Considerable progress has been made in this direction in different laboratories; the project data are accessible through http://wheat.pw.usda.gov/SNP/snpdb.html. In May 2006, the database contained 17174 primers (forward and reverse), 1102 wheat polymorphic loci, and 2224 polymorphic sequence tagged sites in diploid ancestors of polyploid wheat. Zhang et al. [140] also reported 246 gene loci with SNPs and/or small insertions/deletions from wheat homoeologous group 5. Another set of 101 SNPs (1SNP/212 bp) was discovered from genomic sequence analysis in 26-bread wheat lines and one synthetic line (http://urgi.versailles.inra.fr/GnpSNP/, [141]).

## 6. BAC/BIBAC RESOURCES

BAC/BIBAC libraries have been produced in diploid, tetraploid, and hexaploid wheats (see Table 4). Chromosome-specific BAC libraries were also prepared in hexaploid wheat [142–144]. These BAC resources proved useful for a variety of studies including map-based cloning (see Table 3), organization of wheat genome into gene-rich and gene-poor regions that are loaded with retroelements [8, 145–147], and for physical mapping and sequencing of wheat genome (http://wheatdb.ucdavis.edu:8080/wheatdb/, [11]).

## 7. GENE DISTRIBUTION IN WHEAT: GENE-RICH AND GENE-POOR REGIONS

Genetic and physical maps of the wheat genome, discussed above, have been utilized for a study of gene distribution within the genome [58, 63, 148]. In order to identify and demarcate the gene-containing regions, 3025 loci including 252 phenotypically characterized genes and 17 quantitative trait loci (QTL) were physically mapped with the help of deletion stocks [149, 150]. It was shown that within the genome, genes are not distributed randomly and that there are gene-rich regions (GRRs) and gene-poor regions (GPRs), not only within the wheat genome, but perhaps in all eukaryotes (for reviews, see [4, 151]).

In wheat genome, 48 GRRs containing 94% of gene markers were identified with an average of ∼7 such GRRs (range 5–8) per homoeologous group. It was also shown that different wheat chromosomes differed for number and location of GRRs, with 21 GRRs on the short arms containing 35% of the wheat genes, and the remaining 27 GRRs on the long arms containing about 59% of the genes. The GRRs also vary in their size and in gene-density with a general trend of increased gene-density toward the distal parts of individual chromosome arms. This is evident from the fact that more than 80% of the total marker loci were mapped in the distal half of the chromosomes and ∼58% mapped in the distal 20%.

Among 48 GRRs, there were 18 GRRs (major GRRs), which contained nearly 60% of the wheat genes, covering only 11% of the genome, suggesting a very high density of genes in these GRRs, although the number and density of genes in these 18 GRRs was also variable [149, 150]. It has also been shown that the size of GRRs decreases and the number of GRRs increases, as the genome size increases from rice to wheat [4]. For instance, the average size of gene clusters in rice is ∼300 kb as compared to less than 50 kb in wheat and barley. However, no correlation was observed between the chromosome size and the proportion of genes or the size of the GRRs. For instance, group 3 has the longest chromosomes among the wheat homoeologous groups but contained only 13% of the genes compared to group 5 chromosomes that contained 20% of genes [150].

For the chromosomes of homoeologous group 1, the distribution of genes and recombination rates have been studied in a relatively greater detail. Each chromosome of this group (1A, 1B, 1D) has eight GRRs (ranging in size from 3 Mb to 35 Mb), occupying ∼119 Mb of the 800-Mb-long chromosome. Using this homoeologous group, it was confirmed that the GRRs differ in the number of genes and gene-density even within a chromosome or its arms. For instance, the “1S0.8 region” is the smallest of all GRRs, but has the highest gene-density, which is ∼12 times that in the “1L1.0 region.”

The distribution of GRRs has also been compared with the distribution of chromosome breaks involved in the generation of deletion stocks that are currently available and have been used for physical mapping of wheat genome. It was found that the breakpoints are nonrandom, and occur more frequently around the GRRs (one break every 7 Mb; [58, 67]); they seem to occur around GRRs twice as frequently as one would expect on random basis (one break every 16 Mb; [149]). Consequently, GRRs interspersed by <7-Mb-long GPRs will not be resolved and better resolution would be needed to partition the currently known GRRs into mini-GRRs and GPRs.

It has also been inferred that perhaps in eukaryotic genomes, the “gene-poor” regions preferentially enlarged during evolution, as is obvious in wheat, where large, essentially, “gene-empty” blocks of up to ∼192 Mb are common. Taking polyploidy into account, 30% gene-rich part of the genome is still ∼4 times larger than the entire rice genome [149]. Therefore, gene distribution within the currently defined GRRs of wheat would probably be similar to that in the rice genome, except that the gene-clusters would be smaller and the interspersing “gene-empty” regions would be larger, similar to barley as described above. It has also been shown that the “gene-empty” regions of the higher eukaryotic genomes are mainly comprised of retrotransposons and pseudogenes [152, 153]. The proportion of retrotransposons is significantly higher than pseudogenes, especially in the larger genomes, like those of maize and bread wheat.

## 8. VARIABLE RECOMBINATION RATES

The recombination rate has also been recently shown to vary in different regions of the wheat genome. This was demonstrated through a comparison of consensus physical and genetic maps involving 428 common markers [149, 150]. Recombination in the distal regions was generally found to be much higher than that in the proximal half of individual chromosomes, and a strong suppression of recombination was observed in the centromeric regions. Recombination rate among GRRs present in the distal half of the chromosome was highly variable with higher recombination in some proximal GRRs than in the distal GRRs [149, 150]. The gene poor-regions accounted for only ∼5% of recombination.

It has also been reported that the distribution of recombination rates along individual chromosomes is uneven in all eukaryotes studied so far (for more references, see [154, 155]). Among cereals, the average frequency of recombination in rice (with the smallest genome) is translated into a genetic distance of about 0.003 cM per kb with a range of 0 to 0.06 cM per kb (http://rgp.dna.affrc.go.jp/Publicdata.html) and that of wheat (the largest genome) is 0.0003 cM per kb with a range from 0 to 0.007 cM per kb. Non-recombinogenic regions were observed in yeast as well as in rice, but the highest recombination rate for a region appears to be ∼35-fold lower in rice and 140-fold lower in bread wheat (relative to yeast). It may be due to differences in the resolution of recombination rates, which is ∼400 kb in rice (in wheat the resolution is much lower than in rice), whereas the resolution in recombination hotspots in yeast may be as high as only <1 kb in length. Due to averaging over larger regions, recombination in hotspots in rice and wheat may appear to be low relative to that in yeast [4, 150, 151, 156].

## 9. FLOW CYTOGENETICS AND MICRODISSECTION OF CHROMOSOMES IN WHEAT

Flow cytogenetics and microdissection facilitated physical dissection of the large wheat genome into smaller and defined segments for the purpose of gene discovery and genome sequencing. Flow karyotypes of wheat chromosomes were also prepared [157–159]. DNA obtained from the flow-sorted chromosomes has been used for the construction of chromosome-specific large-insert DNA libraries, as has been done for chromosome 4A [157, 159]. Later, all individual 42 chromosome arms involving 21 wheat chromosomes were also sorted out using flow cytometry [160]. In another study, it was also possible to microdissect 5BL isochromosomes from meiotic cells and to use their DNA with degenerate oligonucleotide primer PCR (DOP-PCR) to amplify chromosome arm-specific DNA sequences. These amplified PCR sequences were then used as probes for exclusive painting of 5BL [161].

Flow sorting in wheat has also been used for efficient construction of bacterial artificial chromosome (BAC) libraries for individual chromosomes [143, 162]. The use of these chromosome- and chromosome arm-specific BAC libraries is expected to have major impact on wheat genomics research [1]. For instance, the availability of 3B-specific BAC library facilitated map-based cloning of agronomically important genes such as major QTL for Fusarium head blight resistance [98]. Flow cytometry can also be used to detect numerical and structural changes in chromosomes and for the detection of alien chromosomes or segments thereof (reviewed in detail by [163]). For instance, a 1BL.1RS translocation could be detected by a characteristic change in the flow karyotype [164]. In addition, DNA from flow-sorted chromosomes can be used for hybridization on DNA arrays and chips, with the aim of assigning DNA sequences to specific chromosome arms. This technique will be extensively used now with the availability of Affymetrix wheat GeneChip [165].

## 10. WHEAT GENE SPACE SEQUENCING

International Triticeae Mapping Initiative (ITMI), at its meeting held at Winnipeg, Canada during June 1–4, 2003, took the first initiative toward whole genome sequencing (WGS) in wheat and decided to launch a project that was described as International Genome Research of Wheat (IGROW) by B. S. Gill. A workshop on wheat genome sequencing was later organized in Washington, DC during November 11–13, 2003, which was followed by another meeting of IGROW during the National Wheat Workers Workshop organized at Kansas, USA, during Feb 22–25, 2004 [166]. Consequently, IGROW developed into an International Wheat Genome Sequencing Consortium (IWGSC). Chinese Spring (common wheat) was selected for WGS, since it already had ample genetic and molecular resources [1].

Three phases were proposed for sequencing the wheat genome: pilot, assessment, and scale up. The first phase was recommended for 5 years and is mainly focused on the short-term goal of IWGSC, involving physical and genetic mapping along with sample sequencing of the wheat genome aimed at better understanding of the wheat genome structure. The assessment phase will involve determining which method(s) can be used in a cost-effective manner to generate the sequence of the wheat genome. After a full assessment, the scale-up phase will involve the deployment of optimal methods on the whole genome, obtaining the genome sequence and annotation, which is the long-term goal of IWGSC. With the availability of new sequencing technologies provided by 454/Roche and those provided by Illumina/Solexa and ABI SOLiD [167]; sequencing of gene space of the wheat genome, which was once thought to be almost impossible, should become possible within the foreseeable future.

First pilot project for sequencing of gene space of wheat genome, led by INRA in France, was initiated in 2004 using the largest wheat chromosome, 3B (1GB = 2x the rice genome) of hexaploid wheat as a model. As many as 68000 BAC clones from a 3B chromosome specific BAC library [143] were fingerprinted and assembled into contigs, which were then anchored to wheat bins, covering ∼80% of chromosome 3B. Currently, one or more of these contigs are being sequenced [11], which will demonstrate the feasibility of large-scale sequencing of complete gene space of wheat genome.

## 11. FUNCTIONAL GENOMICS

The determination of the functions of all the genes in a plant genome is the most challenging task in the postgenomic era of plant biology. However, several techniques or platforms, like serial analysis of gene expression (SAGE), massively parallel signature sequencing (MPSS), and micro- and macroarrays, are now available in several crops for the estimation of mRNA abundance for large number of genes simultaneously. The microarrays have also been successfully used in wheat for understanding alterations in the transcriptome of hexaploid wheat during grain development, germination and plant development under abiotic stresses [168, 169]. Recently, a comparison was made between Affymetrix GeneChip Wheat Genome Array (an in-house custom-spotted complementary DNA array) and quantitative reverse transcription-polymerase chain reaction (RT-PCR) for the study of gene expression in hexaploid wheat [170]. Also, functional genomics approach in combination with “expression genetics” or “genetical genomics” provides a set of candidate genes that can be used for understanding the biology of a trait and for the development of perfect or diagnostic marker(s) to be used in map-based cloning of genes and MAS [9]. A similar example was provided by Jordan et al. [9], when they identified regions of wheat genome controlling seed development by mapping 542 eQTLs, using a DH mapping popultion that was earlier used for mapping of SSRs and QTL analysis of agronomic and seed quality traits [171]. Expression analysis using mRNA from developing seeds from the same mapping population was also conducted using Affymetrix GeneChip Wheat Genome Array [172].

### 11.1. RNA interference for wheat functional genomics

RNA interference (RNAi), which was the subject of the 2006 Nobel Prize in Physiology or Medicine, is also being extensively utilized for improvement of crop plants [173]. This technique does not involve introduction of foreign genes and thus provides an alternative to the most controversial elements of genetic modification. Plans in Australia are underway, where the knowledge gained from RNAi approach will be used for developing similar wheats by conventional method of plant breeding, as suggested by CSIRO scientists for developing high-fibre wheat [174]. In bread wheat, in particular, the technology provides an additional advantage of silencing all genes of a multigene family including homoeoloci for individual genes, which are often simultaneously expressed, leading to a high degree of functional gene redundancy [175]. It has been shown that delivery of specific dsRNA into single epidermal cells in wheat transiently interfered with gene function [176, 177]. Yan et al. [90] and Loukoianov et al. [178] used RNAi for stable transformation and to demonstrate that RNAi-mediated reduction of *VRN2* and *VRN1* transcript levels, respectively, accelerated and delayed flowering initiation in winter wheat. Similarly, Regina et al. [179] used RNAi to generate high-amylose wheat. However, none of the above studies reported long-term phenotypic stability of RNAi-mediated gene silencing over several generations, neither did they report any molecular details on silencing of homoeologous genes. However, Travella et al. [180] showed RNAi results in stably inherited phenotypes suggesting that RNAi can be used as an efficient tool for functional genomic studies in polyploid wheat. They introduced dsRNA-expressing constructs containing fragments of genes encoding *Phytoene Desaturase* (*PDS*) or the signal transducer of ethylene, *Ethylene Insensitive 2* (*EIN2*) and showed stably inherited phenotypes of transformed wheat plants that were similar to mutant phenotypes of the two genes in diploid model plants. Synthetic microRNA constructs can also be used as an alternative to large RNA fragments for gene silencing, as has been demonstrated for the first time in wheat by Yao et al. [181] by discovering and predicting targets for 58 miRNAs, belonging to 43 miRNA families (20 of these are conserved and 23 are novel to wheat); more importantly four of these miRNAs are monocot specific. This study will serve as a foundation for the future functional genomic studies. The subject of the use of RNAi for functional genomics in wheat has recently been reviewed [173].

### 11.2. TILLING in wheat

Recently, Targeting Induced Local Lesions IN Genomes (TILLING) was developed as a reverse genetic approach to take advantage of DNA sequence information and to investigate functions of specific genes [182]. TILLING was initially developed for model plant *Arabidopsis thaliana* [183] having fully sequenced diploid genome and now has also been successfully used in complex allohexaploid genome of wheat, which was once considered most challenging candidate for reverse genetics [184].

To demonstrate the utility of TILLING for complex genome of bread wheat, Slade et al. [185] created TILLING library in both bread and durum wheat and targeted *waxy* locus, a well characterized gene in wheat encoding granule bound *starch synthase I* (*GBSSI*). Loss of all copies of this gene results in the production of *waxy* starch (lacking amylose). Production of waxy wheat by traditional breeding was difficult due to lack of genetic variation at one of the *waxy* loci. However, targeting *waxy* loci by TILLING [185], using locus specific PCR primers led to identification of 246 alleles (196 alleles in hexaploid and 50 alleles in tetraploid) using 1920 cultivars of wheat (1152 hexaploid and 768 tetraploid). This made available novel genetic diversity at *waxy* loci and provided a way for allele mining in important germplasm of wheat. The approach also allowed evaluation of a triple homozygous mutant line containing mutations in two waxy loci (in addition to a naturally occurring deletion of the third locus) and exhibiting a near waxy phenotype.

Another example of on-going research using TILLING in wheat is the development of EMS mutagenised populations of *T. aestivum* (cv. Cadenza, 4200 lines, cv. Paragon, 6000 lines), *T. durum* (cv. Cham1, 4,200 lines), and *T. monococcum* (Accession DV92, 3000 lines) under the Wheat Genetic Improvement Network (WGIN; funded by Defra and BBSRC in the UK and by the EU Optiwheat programme). The aim of this program is to search noval variant alleles for *Rht-b1c,RAR-1, SGT-1*, and *NPR-1* genes (personal communication: andy.phillips@bbsrc.ac.uk and Simon.Orford@bbsrc.ac.uk).

The above examples provide proof-of-concept for TILLING other genes, whose mutations may be desired in wheat or other crops. However, homoeolog-specific primers are required in order to identify new alleles via TILLING in wheat. In case of *waxy*, the sequences of the three homoeologous sequences were already known, which facilitated primer designing, but TILLING of other genes may require cloning and sequencing of these specific genes in order to develop homoeolog-specific target primers.

## 12. COMPARATIVE GENOMICS

In cereals, a consensus map of 12 grass genomes including wheat is now available, representing chromosome segments of each genome relative to those in rice on the basis of mapping of anchor DNA markers [186]. Some of the immediate applications of comparative genomics in wheat include a study of evolution [187] and isolation/characterization of genes using the model genome of rice. The genes, which have been examined using comperative genomics approach include the pairing gene, *Ph1* [102, 188], gene(s) controlling preharvest sprouting (PHS; [189]), receptor-like kinase loci [190], gene for grain hardness [191], genes for glume coloration and pubescence (*Bg, Rg*; [192]), and the *Pm3* gene, responsible for resistance against powdery mildew [187].

**Conservation of colinearity and synteny**

Among cereals, using molecular markers, colinearity was first reported among A, B, and D subgenomes of wheat [13, 193], and later in the high-gene density regions of wheat and barley. At the *Lrk10* locus in wheat and its orthologous region in barley, a gene density of one gene per 4-5 kb was observed, which was similar to that found in *A. thaliana* [6]. Conservation of colinearity between homoeologous A genomes of diploid einkorn wheat and the hexaploid was also exploited for chromosome walking leading to cloning of candidate gene for the leaf rust resistance locus *Lr10* in bread wheat [194]. *Lr10* locus along with LMW/HMW loci of diploid wheat, when compared with their orthologs from tetraploid and hexaploid wheats, was found to be largely conserved except some changes that took place in intergenic regions [195–197]. On the basis of divergence of intergenic DNA (mostly transposable elements), tetraploid and hexaploid wheats were shown to have diverged about 800 000 years ago [197]. Similarly, the divergence of diploid from the tetraploid/hexaploid lineage was estimated to have occurred about 2.6–3 million years ago [195, 196].

Notwithstanding the above initial demonstration of colinearity using molecular markers, later studies based on genome sequences suggested disruption of microcolinearity in many regions thus complicating the use of rice as a model for cross-species transfer of information in these genomic regions. For instance, Guyot et al. [198] conducted an in silico study and reported a mosaic conservation of genes within a novel colinear region in wheat chromosome 1AS and rice chromosome 5S. Similarly, Sorrells et al. [199] while comparing 4485 physically mapped wheat ESTs to rice genome sequence data belonging to 2251 BAC/PAC clones, resolved numerous chromosomal rearrangements. The above findings also received support from sequence analysis of the long arm of rice chromosome 11 for rice-wheat synteny [200].

More recently, the grass genus *Brachypodium* is emerging as a better model system for wheat belonging to the genus *Triticum*, because of a more recent divergence of these two genera (35–40 million years) relative to wheat-rice divergence [201–203]. Also, sequence of *Brachypodium,* which is likely to become available in the near future, may help further detailed analyses of colinearity and synteny among grass genomes. This has already been demonstrated through a comparison of 371 kb sequence of *B. sylvaticum* with orthologous regions from rice and wheat [204]. In this region, *Brachypodium* and wheat showed perfect macrocolinearity, but rice was shown to contain ∼220 kb inversion relative to *Brachypodium* sequence. Also, in *Ph1* region, more orthologous genes were identified between the related species *B. sylvaticum* and wheat than between wheat and rice, thus once again demonstrating relative utility of *Brachypodium* genome as a better model than rice genome for wheat comparative genomics [102, 188].

## 13. EPIGENETICS IN WHEAT

Epigenetics refers to a heritable change that is not a result of a change in DNA sequence, but, instead, results due to a chemical modification of nucleotides in the DNA or its associated histone proteins in the chromatin. Several studies have recently been intiated to study the epigenetic modifications in the wheat genome. For instance, methylation-sensitive amplified polymorphism (MSAP) has been used to analyze the levels of DNA methylation at four different stages (2d, 4d, 8d, and 30d after pollination) of seed development in bread wheat [205]. It was found that 36–38% of CCGG sites were either fully methylated at the internal C’s and/or hemimethylated at the external C’s at the four corresponding stages. Similarly, Shitsukawa et al. [206] also studied genetic and epigenetic alterations among three homoeologs in the two class E-type wheat genes for flower development, namely, *wheat SEPALLATA* (*WSEP*) and *wheat LEAFY HULL STERILE1* (*WLHS1*). Analyses of gene structure, expression patterns, and protein functions showed that no alterations were present in the *WSEP* homoeologs. By contrast, the three *WLHS1* homoeologs showed genetic and epigenetic alterations. It was shown that *WLHS1-B* was predominantly silenced by cytosine methylation, suggesting that the expression of three homoeologous genes is differentially regulated by genetic or epigenetic mechanisms. Similar results were reported for several other genes like *TaHd1* involved in photoperiodic flowering pathway, *Ha* for grain hardness, and *TaBx* for benzoxazinone biosynthesis [207–209].

A prebreeding program in wheat (along with barley and canola) based on epigenetically modified genes has also been initiated in Australia at CSIRO, under the leadership of Dr. Liz Dennis and Dr. Jim Peacock, with the support from Dr. Ben Trevaskis (http://www.grdc.com.au/director/events/groundcover?item_id=A5B55D1DED8B9C20860C0CDE8C6EE077&article_id=A97C28B1F1614E34835D6BDB8CBDC75C). This pioneering work will involve vernalization, the mechanism that allows winter crops to avoid flowering until spring, when long days and mild conditions favor seed setting and grain filling. They plan to breed varieties with a wider range of heading dates and improved frost tolerance during flowering. In wheat (as also in other cereals), the epigenetic component is also built around *VRN1* gene, which plays a role analogous to that of *Flowering Locus C* (*FLC*) in *Arabidopsis* and canola. *VRN1* is one of the most important determinants of heading dates in winter cereals including wheat and also accounts for difference between winter and spring wheat varieties. It has been shown that during vegetative growth, *VRN1* is repressed epigenetically; this repression is lifted in spring, allowing the protein encoded by *VRN1* to activate other genes involved in reproduction. As many as ∼3000 wheat varieties are being looked at for variation in their *VRN1* gene so as to breed better combinations of heading date and frost tolerance (http://www.grdc.com.au/director/events/groundcover?item_id=A5B55D1DED8B9C20860C0CDE8C6EE077&article_id=A97C28B1F1614E34835D6BDB8CBDC75C).

**Wheat allopolyploidy and epigenetics**

Polyploidization induces genetic and epigenetic modifications in the genomes of higher plants including wheat (reviewed in [210, 211]). Elimination of noncoding and low-copy DNA sequences has been reported in synthetic allopolyploids of *Triticum* and *Aegilops* species [212–214]. In two other studies, patterns of cytosine methylation were also examined throughout the genome in two synthetic allotetraploids, using methylation-sensitive amplification polymorphism (MSAP; [215, 216]). This analysis indicated that the parental patterns of methylation were altered in the allotetraploid in 13% of the genomic DNA analyzed. Gene silencing and activation were also observed when 3072 transcribed loci were analyzed, using cDNA-AFLP [217, 218]. This study demonstrated new, nonadditive patterns of gene expression in allotetraploid, as indicated by the fact that 48 transcripts disappeared and 12 transcripts that were absent in the diploid parents, appeared in the allotetraploid. These results were found reproducible in two independent synthetic allotetraploids. The disappearance of transcripts could be related to gene silencing rather than gene loss and was partly associated with cytosine methylation. In another similar study involving artificially synthesized hexaploid wheats and their parents, down-regulation of some genes and activation of some other genes, selected in a nonrandom manner, was observed [219]. The genome-wide genetic and epigenetic alterations triggered by allopolyploidy thus suggested plasticity of wheat genome. The reproducibility of genetic and epigenetic events indicated a programmed rather than a chaotic response and suggests that allopolyploidy is sensed in a specific way that triggers specific response rather than a random mutator response [218].

## 14. QUANTITATIVE TRAIT LOCI (QTL) AND PROTEIN QUANTITATIVE LOCI (PQLs) IN WHEAT

A large number of QTL studies for various traits have been conducted in bread wheat, leading to mapping of QTL for these traits on different chromosomes. In most of these studies, either single marker regression approach or QTL interval mapping has been utilized. Although most of these studies involved mapping of QTL with main effects only, there are also reports of QTL, which have no main effects but have significant digenic epistatic interactions and QTL × environment interactions [220–222]. A detailed account of studies involving gene tagging and QTL analyses for various traits conducted in wheat is available elsewhere [14, 223]. More up-to-date accounts on QTL studies (summarized in Table 5) are also available for disease resistance [224], for resistance against abiotic stresses [225], grain size, and grain number [226], and for several other traits including yield and yield contributing characters, plant type, and flowering time [222, 227]. Advanced backcross QTL (AB-QTL) analysis, proposed by Tanksley and Nelson [228], has also been utilized in wheat to identify QTL for a number of traits including yield and yield components, plant height, and ear emergence [129, 229]. More recently AB-QTL analysis was practiced for the identification of QTL for baking quality traits in two BC_2_F_3_ populations of winter wheat [230].

Quantitative variation in protein spots was also used for detection of protein quantitative loci (PQL) in wheat. For instance, in a study, 170-amphiphilic protein spots that were specific to either of the two parents of ITMIpop were used for genotyping 101 inbred lines; 72 out of these 170 proteins spots were assigned to 15 different chromosomes, with highest number of spots mapped to Group-1 chromosomes. QTL mapping approaches were also used to map PQL; 96 spots out of the 170 specific ones showed at least one PQL. These PQL were distributed throughout the genome. With the help of MALDI-TOF spectrometry and database search, functions were also assigned to 93 specific and 41 common protein spots. It was shown in the above study that majority of these proteins are associated with membranes and/or play a role in plant defense against external invasions [231].

### 15. Recent insights into the origin/evolution of wheat genomes

**Figure 1:**
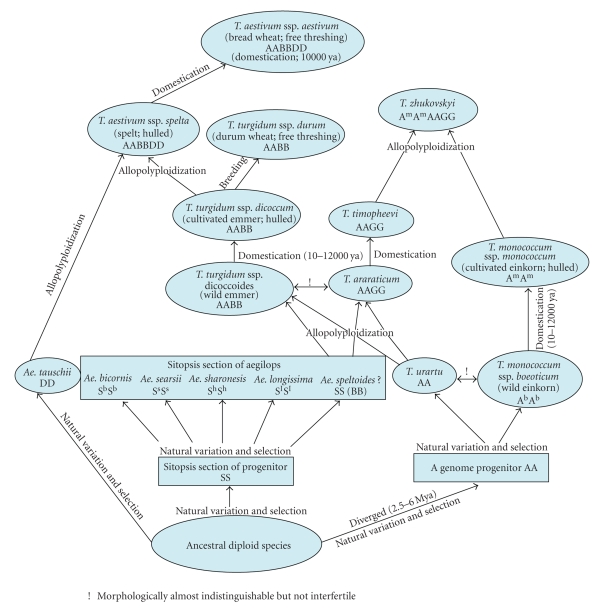
Schematic representation of the evolutionary history of wheat species (*Triticum* and *Aegilops*).

In the genomics era, the subject of origin and evolution of bread wheat has also been revisited. This gave new insights into the identity of progenitors of the three subgenomes (A, B, D) of bread wheat, and into the genome alterations, which presumably accompanied the course of its evolution and domestication (see Figure 1). These aspects of evolution of bread wheat will be discussed briefly in this section.

### 15.1. Origin of A, B, and D subgenomes

As mentioned earlier, bread wheat is a segmental allohexaploid having three closely related subgenomes A, B, and D. Initial analysis of the three subgenomes of bread wheat was mainly based on studies involving chromosome pairing in interspecific hybrids, and karyotype analysis in bread wheat as well as in the probable donors of the subgenomes (for reviews, see [232–236]). However, more recently, molecular markers and DNA sequence data have been used for the analysis of these subgenomes (see [237–239]). As a result, we have known with some degree of certainty that *T*. *urartu* (2 *n* = 14) is the donor of subgenome A and *Ae*. *tauschii* (synonyms, *T. tauschii, Ae. squarrosa*) is the donor of subgenome D; this has recently been confirmed through analysis of DNA sequences of two genes, namely, *Acc-1* (plastid acetyl-CoA carboxylase) and *Pgk-1* (plastid 3-phosphoglycerate kinase) [240]. In contrast to this, although *Ae. speltoides* was once considered as the probable donor of the B subgenome ([241], for a review, see [237]), studies carried out later showed that *Ae. speltoides* more closely resembles the subgenome G of *T. timopheevii* rather than to the subgenome B of bread wheat. DNA sequences of the above genes, *Acc-1* and *Pgk-1* also proved to be of no help in identification of the progenitor of the subgenome B. There is, thus still no unanimity on the progenitor of the subgenome B of bread wheat (for more details, see [242]), and there are speculations that the donor of the subgenome B might have lost its identity during evolution and may never be discovered.

DNA sequences of genes other than the above two genes have also been used for the study of origin and evolution of the component subgenomes of bread wheat. For instance, in one such study, sequences from 14 loci (2 sequences from each of the 7 chromosomes) belonging to the subgenome B of bread wheat, when compared with those from five diploid species (from section Sitopsis) closely related to the B subgenome of bread wheat, indicated that the B subgenome of bread wheat and the genomes of the above five diploid species diverged greatly after the origin of tetraploid wheat [243]. The above study also received support from the recent evidence of independent origins of wheat B and G subgenomes [244]. In this study, 70 AFLP loci were used to sample diversity among 480 wheat lines collected from their natural habitats, which encompassed the entire range of habitats for all S genome *Aegilops* species. Also, a comparison of 59 *Aegilops* representatives of S genome diversity with 2x, 4x chromosome number, and 11 nulli-tetrasomic wheat lines at 375 AFLP loci suggested that B genome chromosomes of 6x wheat were derived from chromosomes of *Ae. speltoides*, and no other species. Further, an analysis of the haplotypes at nuclear and chloroplast loci *ACC1*, *G6PDH*, *GPT*, *PGK1*, *Q*, *VRN1*, and *ndhF* for ∼70 *Aegilops* and *Triticum* lines (0.73 Mb sequenced) revealed that both B and G genomes of polyploid wheats are unique samples of *A. speltoides* haplotype diversity. However, it is likely that due to the outbreeding nature of *A. speltoides*, no modern *A. speltoides* lines have preserved the B donor genotype in its ancestral state. The above findings can be incorporated into a broader scheme of wheat genome evolution (see Figure 1) with resolved positions of the B genome relative to S progenitors and G sisters. Similar analysis of the D subgenome and its progenitor showed that the D subgenome had more than one allele for a single locus derived from a progenitor, suggesting that hexaploid wheat perhaps originated from tetraploid wheat more than once utilizing different sources of *Ae. tauschii* [245]. Also, it was realized that major part of the large genome (16000 Mb) of bread wheat is composed of transposable elements (TEs). Therefore, the role of TEs in the evolution of bread-wheat and allied genomes has also been examined [246, 247]. In these studies, some specific sequences from A and B genomes of diploid species were located, respectively, in B- and A-subgenomes of bread wheat, suggesting the role of TEs in transfer of sequences between A and B subgenomes. A bioinformatics approach was also used on a large genomic region (microgenomic approach) sequenced from *T. monococcum* (AA) and *Ae. tauschii* (DD). This approach allowed a comparison of variation within coding regions with that in the noncoding regions of the subgenomes.

### 15.2. Alterations that accompanied domestication

Domestication of most crop plants including wheat involved transition from short day, small-seeded plants with natural seed dispersal to photoperiod insensitive, large-seeded nonshattering plants. A study of genetic loci underlying domestication-related traits in *T. dicoccoides* was also conduced [430], where seven domestication syndrome factors (DSFs) were proposed, each affecting 5–11 traits. Following conclusions were made with respect to the domestication-related QTL. (i) Some of these QTL had strong effect and were clustered. (ii) Strong QTL were mainly associated with GRRs, where recombination rates are high. (iii) These QTL predominantly occurred in the A genome, suggesting that A genome has played a more important role than the B genome in evolution during domestication; this is understandable, because einkorn diploid wheat (*T. monococcum*) carrying the A genome was the first wheat to be domesticated, so that most of the domestication related traits in different wheats must have been selected within the A genome. Similar studies involving study of evolution during domestication were also conducted in hexaploid wheats for seed size, free threshing habit, rachis stiffness, photoperiod insensitivity, and so forth (for a review, see [431]). In wheat, a primary component of domestication syndrome was the loss of spike shattering, controlled by *Br* (brittle rachis) loci on chromosome 3A and 3B [414]. Other traits of wheat domestication syndrome shared by all domesticated wheats are the soft glumes, increased seed size, reduced number of tillers, more erect growth, and reduced dormancy [432]. A gene *GPC-B1*, which is an early regulator of senescence with pleiotropic effects on grain nutrient content, has also been found to affect seed size [96]. However, in some genotypes and environments, the accelerated grain maturity conferred by functional *GPC-B1* allele has been found associated with smaller seeds [433], suggesting that indirect selection for large seeds may explain the fixation of the nonfunctional *GPC-B1* allele in both durum and bread wheats [96]. Among many genes relevant to wheat domestication syndrome, only *Q* and *GPC-B1* have been successfully isolated so far, suggesting a need for systematic effort to clone other genes, since it is possible that genetic variation at these loci might have played an important role in the success of wheat as a modern crop.

## 16. APPLICATION OF GENOMICS TO MOLECULAR BREEDING OF WHEAT

### 16.1. Association mapping in wheat

Association mapping is a high-resolution method for mapping QTL based on linkage disequilibrium (LD) and holds great promise for genetic dissection of complex traits. It offers several advantages, which have been widely discussed [434, 435]. In wheat, some parts of the genome relative to other parts are more amenable to LD/association mapping for QTL detection and fine mapping, since the level of LD is variable across the length of a chromosome. As we know, LD decay over longer distances will facilitate initial association of trait data with the haplotypes in a chromosome region and LD decay over short distances will facilitate fine mapping of QTL [12].

Several studies involving association mapping in wheat have been conducted in the recent past. For instance, association mapping has been conducted for kernel morphology and milling quality [436] and for the quantity of a high-molecular-weight glutenin [141, 437]. In another study, 242 diversity array technology (DArT) markers were utilized for association mapping of genes/QTL controlling resistance against stem rust (SR), leaf rust (LR), yellow rust (YR), powdery mildew (PM), and those controlling grain yield (GY). Phenotypic data from five historical CIMMYT elite spring wheat yield trials (ESWYT) conducted in a large number of international environments were utilized for this purpose and two linear mixed models were applied to assess marker-trait associations after a study of population structure and additive genetic covariance between relatives [438]. A total of 122, 213, 87, 63, and 61 DArT markers were found to be significantly associated with YR, GY, LR, SR, and PM, respectively. Association analysis was also conducted between markers in the region of a major QTL responsible for resistance to *Stagonospora nodorum* (causing glume blotch); it was concluded that association mapping had a marker resolution, which was 390-fold more powerful than QTL analysis conducted using an RIL mapping population [439]. Such high-resolution mapping of traits and/or QTL to the level of individual genes, using improved statistical methods, will provide new possibilities for studying molecular and biochemical basis of quantitative trait variation and will help to identify specific targets for crop improvement.

### 16.2. Marker-assisted selection in wheat

A large number of marker-trait associations determined during the last decades facilitated the use of molecular markers for marker-assisted selection (MAS) in bread wheat, which is gaining momentum in several countries. In particular, major programs involving MAS in wheat are currently underway in USA, Australia, and at CIMMYT in Mexico. In USA, a wheat MAS consortium comprosing more than 20 wheat-breeding programs was constituted at the end of 2001. The objective of this consortium was to apply and to integrate MAS in public wheat breeding programs [440]. Under these programs, MAS has been utilized for transfer of as many as 27 different insect and pest resistance genes and 20 alleles with beneficial effects on bread making and pasta quality into ∼180 lines adapted to the primary US production regions. These programs led to release of germplasm consisting of 45 MAS-derived lines [441]. Similarly, the program in Australia involved improvement of 20 different traits (including resistance to some abiotic stresses) and has already led to release of some improved cultivars ([442], Peter Langridge personal communication). Among these traits, MAS has become a method of choice for those agronomically important traits, where conventional bioassays were expensive and unconvincing, as was the case in selection for cereal cyst nematodes resistance carried out by Agriculture Victoria [443]. In addition to this, MAS has been incorporated in backcross breeding in order to introgress QTL for improvement of transpiration efficiency and for negative selection for undesirable traits such as yellow flour color [444]. Australian scientists also conducted a computer simulation in order to design a genetically effective and economically efficient marker-assisted wheat-breeding strategy for a specific outcome. This investigation involved an integration of both restricted backcrossing and doubled haploid (DH) technology. Use of MAS at the BC_1_F_1_ followed by MAS in haploids derived from pollen of BC_1_F_1_ (prior to chromosome doubling) led to reduction of cost of marker-assisted breeding up to 40% [445]. Later, this MAS strategy was validated practically in a marker-assisted wheat-breeding program in order to improve quality and resistance against rust disease (for review, see [446]). At CIMMYT, markers associated with 25 different genes governing insect pest resistance, protein quality, homoeologous pairing, and other agronomic characters are currently being utilized in wheat breeding programs in order to develop improved wheat cultivars [447]. Some of the markers used in these programs are perfect markers that have been developed from available nucleotide sequences of these genes. In future, large-scale sequencing of GRRs (gene-rich regions), to be undertaken by IWGSC, will also facilitate isolation of important genes for production of improved transgenic crops, and for development of “perfect markers” for agronomically important traits to be used in MAS [448, 449].

## 17. ORGANELLAR GENOMES AND THEIR ORGANIZATION

The genomes of wheat chloroplast and mitochondrion have also been subjected to a detailed study during the last decade. The results of these studies will be briefly discussed in this section.

### 17.1. Chloroplast genome

In bread wheat, 130–155 chloroplasts, each containing 125–170 circular DNA molecules (135 kb), are present in each mesophyll cell, thus making 16000–26000 copies of cpDNA within a cell. This makes 5–7% of the cellular DNA in the leaf and 10–14% of the DNA in a mesophyll cell. In the related diploid species, there are 4900–6600 copies and in tetraploid species, there are 9600–12400 copies of cpDNA per mesophyll cell.

The wheat chloroplast genome, like all other plant chloroplast genomes, has two inverted repeat regions, each copy (21-kb-long) separated from the other by two single copy regions (12.8 kb, 80.2 kb). The gene content of wheat chloroplast is the same as those of rice and maize plastomes, however some structural divergence was reported in the gene coding regions, due to illegitimate recombination between two short direct repeats and/or replication slippage; this included the presence of some hotspot regions for length mutations. The study of deletion patterns of open reading frames (ORFs) in the inverted-repeat regions and in the borders between the inverted repeats and the small single-copy regions supports the view that wheat and rice are related more closely to each other than to maize (see [450, 451]). Deletions, insertions, and inversions have also been detected during RFLP analysis of cpDNA, which gave eleven different cpDNA types, in the genus *Triticum*, the bread wheat sharing entirely the cpDNA type with durum wheats, but not with that of any of the diploid species. The cpDNA of *Ae. speltoides* showed maximum similarity to those of *T. aestivum*, *T. timopheevii*, and *T. zhukovskyi*, suggesting that *Ae. speltoides* should be the donor of the B subgenome of common wheat [452].

### 17.2. Mitochondrial genome

Wheat mtDNA is larger (430 kb) than cpDNA (135 kb) with a minimum of 10 repeats but encodes only 30–50% polypeptides relative to cpDNA. Thus, large amount of mtDNA is noncoding, there being about 50 genes involved in RNA synthesis [453]. Mitochondrial genome of Chinese Spring has been sequenced using 25 cosmid clones of mitochondrial DNA, selected on the basis of their gene content. This led to the identification of 55 (71) genes including the following: 18 genes (20) for electron transport system, 4 genes for mitochondrial biogenesis, 11 genes for ribosomal proteins, 2 genes for splicing and other function, 3 genes (10) for rRNAs, and 17 genes (24) for tRNAs (the numerals in parentheses represent number of genes, taking multiple copies of a gene as separate genes). When mitochondrial gene maps were compared among wheat, rice, and maize, no major synteny was found between them other than a block of two to five genes. Therefore, mitochondrial genes seem to have thoroughly reshuffled during speciation of cereals. In contrast, chloroplast genes show perfect synteny among wheat, rice, and maize [451].

## 18. CONCLUSIONS

Significant progress during the last two decades has been made in different areas of wheat genomics research. These include development of thousands of molecular markers (including RFLPs, SSRs, AFLPs, SNPs, and DArT markers), construction of molecular genetic and physical maps (including radiation hybrid maps for some chromosomes) with reasonably high density of markers, development of more than 1 million ESTs and their use for developing functional markers, and the development of BAC/BIBAC resources for individual chromosomes and entire subgenomes to facilitate genome sequencing. Functional genomics approaches like TILLING, RNAi, and epigenetics have also been utilized successfully, and a number of genes/QTL have been cloned to be used in future wheat improvement programs. Organellar genomes including chloroplast and mitochondrial genomes have been fully sequenced, and we are at the threshold of initiating a major program of sequencing the gene space of the whole nuclear genome in this major cereal. The available molecular tools also facilitated a revisit of the wheat community to the problem of origin and evolution of the wheat genome and helped QTL analysis (including studies involving LD and association mapping) for identification of markers associated with all major economic traits leading to the development of major marker-aided selection (MAS) programs for wheat improvement in several countries.

## Figures and Tables

**Table 1:** A list of some important molecular maps developed in wheat.

Map type/class of wheat	Population used for mapping	No. of loci mapped	Genetic map length (cM)	Reference
RFLP maps				
Diploid wheat (D-genome)	F_2_ [*T. tauschii* (TA1691 var. meyeri × TA1704 var. typica)]	152	1554	[25]
Diploid wheat (D-genome)	F_2_ [*Aegilops tauschii* var. meyeri(TA1691) ×*Ae. tauschii* var. typica(TA 1704)]	546	—	[26]

SSR maps				
Bread wheat	ITMI RILs (W7984 × Opata85)	279	—	[18]
Bread wheat	RILs (Synthetic × Opata)	1235	2569	[16]
Bread wheat	RILs (W7984 × Opata85)	1406	2654	[27]
Bread wheat	DHs (Kitamoe × Munstertaler)	464	3441	[28]
Bread wheat*	RILs (Chuan-Mai18 × Vigour18)	244	3150	[29]

AFLP maps				
Bread wheat*	RILs (Wangshuibai × Alondra’s)	250	2430	[30]

Composite maps				
Einkorn wheat	F_2s_/F_3s_ (*T. monococcum*ssp *. monococcum* DV92 × *T. monococcum* ssp. *aegilopoides* C3116) (marker loci-mainly RFLPs)	3335	714	[31]
Einkorn wheat	RILs (*Triticum boeoticum* × *T. monococcum*) marker loci-RFLPs, SSR	177	1262	[5]
Durum wheat	RILs (*T. durum* var. Messapia × *T. turgidium* var. MG4343) (marker loci-RFLP, Glu3B, others)	213	1352	[32]
Durum wheat	RILs (*T. durum* var. Messapia × *T. turgidium* var. MG4343) (marker loci-AFLPs, RFLPs)	88	2063	[33]
Durum wheat	RILs (Jennah Khetifa × Cham10 (marker loci-RFLPs, SSRs, AFLPs)	206	3598	[34]
Durum wheat*	RILs (Omrabi 5 × *T dicoccoides* 600545) (marker loci-SSRs, AFLPs)	312	2289	[35]
Bread wheat	RILs (*T. aestivum* L. var. Forno × *T. spelta* L. var. Oberkulmer) (marker loci-RFLPs, SSRs)	230	2469	[36]
Bread wheat*	DHs (CM-82036 × Remus) (marker loci-RFLPs, AFLPs, SSRs, etc.)	384	1860	[37]
Bread wheat*	DHs (Savannah × Senat) (marker loci-SSRs, AFLPs)	345 (17)	2300	[38]
Bread wheat*	RILs (Renan × Récital) (marker loci-SSRs, RFPLs, AFLPs)	265 (17)	2722	[39, 40]
Bread wheat	F_5s_(Arina × Forno) (marker loci-RFLPs, SSRs)	396	3086	[41]
Bread wheat	DHs (Courtot × Chinese Spring) (marker loci-RFLPs, SSRs, AFLPs)	659	3685	[42]
Bread wheat*	DHs (Frontana × Remus) (marker loci-SSRs, STSs, AFLPs, etc.)	535	2840	[43]
Bread wheat	RILs (Grandin × BR34) (marker loci-TRAPs, SSRs)	352	3045	[44]
Bread wheat*	DHs (Spring × SQ1) (marker loci-AFLPs, SSRs)	567	3521	[45]
Bread wheat*	RILs (Dream × Lynx) (marker loci-SSRs, STSs, AFLPs)	283 (17)	1734	[46]
Bread wheat*	DHs (AC Karma × 87E03-S2B1) (marker loci-STSs, SSRs, etc.)	167 (15)	2403	[47]
Bread wheat*	DHs (Trident × Molineux) (marker loci-SSRs, STSs, RFLPs, etc.)	251	3061	[48]
Bread wheat*	DH (Arina × Riband) (marker loci-AFLPs, SSRs)	279	1199	[49]
Bread wheat*	DHs (RL4452 × AC Domain) (marker loic-SSRs, genes, etc.)	369	2793	[50]
Bread wheat*	RILs (Chuan 35050 × Shannong 483) (marker loci-SSRs, EST-SSRs, ISSRs, SRAPs,TRAPs, Glu loci)	381	3636	[51]
Bread wheat*	DHs (Shamrock × Shango) (marker loci-SSRs, DArTs)	263	1337	[52]
Bread wheat	DHs Cranbrook × Halberd (Marker loci-SSRs, RFLPs, AFLPs, DArTs, STSs)	749	2937	[53]

*These are framework linkage map prepared for QTL analyses.

**Table 2:** Deletion-based physical maps of common wheat.

Homoeologous group/	Marker loci	No. of deletion	Reference
chromosome/arm	mapped	stocks used	
1	19 RFLPs	18	[57]
1	50 RFLPs	56	[58]
2	30 RFLPs	21	[59]
2	43 SSRs	25	[60]
3	29 RFLPs	25	[61]
4	40 RFLPs	39	[62]
5	155 RFLPs	65	[63]
5	245 RFLPs, 3 SSRs	36	[64]
5S	100 RFLPs	17	[65]
5A	22 RFLPs	19	[66]
6	24 RFLPs	26	[67]
6	210 RFLPs	45	[68]
6S	82 RFLPs	14	[69]
7	16 RFLPs	41	[70]
7	91 RFLPs, 6 RAPDs	54	[71]
6B, 2D, and 7D	16 SSRs	13	[72]
1BS	24 AFLPs	8	[73]
4DL	61 AFLPs, 2 SSRs, 2 RFLPs	8	[74]
1BS	22 ESTs	2	[75]
Whole genome	725 SSRs	118	[76]
Whole genome	260 BARC	117	[27]
Whole genome	313 SSRs	162	[77]
Whole genome	16000 ESTs	101	http://wheat.pw.usda.gov/NSF/progressmapping.html
Whole genome	266 eSSRs	105	[78]
Whole genome	672 EST-SSRs	101	[79]

**Table 3:** Genes already cloned or likely to be cloned through map-based cloning in wheat.

Gene/QTL	Trait	Reference
*Lr1*	Leaf rust resistance	[85, 86]
*Lr10*	Leaf rust resistance	[87]
*Lr21*	Leaf rust resistance	[88]
*VRN1*	Vernalization response	[89]
*VRN2*	Vernalization response	[90]
*VRN3*	Vernalization response	[91]
*Q*	Free threshing character	[92, 93]
*Pm3b*	Powdery mildew resistance	[94, 95]
*GPC-B1*	High grain protein content	[96, 97]
*Qfhs.Ndsu-3bs*	Fusarium head blight resistance	[98]
*Yr5*	Resistance to stripe rust	[99]
*B*	Boron tolerance	[100]
*Fr2*	Frost resistance	http://www.agronomy.ucdavis.edu/Dubcovsky
*EPS-1*	Flowering time	http://www.agronomy.ucdavis.edu/Dubcovsky
*Tsn1*	Host-selective toxin *Ptr ToxA*	[101]
*Ph1*	Chromosome pairing locus	[102]
*Sr2*	Stem rust resistance	[103]

**Table 4:** BAC libraries available in wheat.

Species (accession)	Coverage	Restriction site	No. of clones (clone size in kb)	Curator
*T. monococcum* (DV92)	5.6 X	*Hin*d III	276000 (115)	J. Dubcovsky
*T. dicoccoides* (Langdon)	5.0 X	*Hin*d III	516000 (130)	J. Dubcovsky
*T. urartu* (G1812)	4.9 X	*Bam*H I	163200 (110)	J. Dvorak
*Ae. tauschii* (AL8/78)	2.2 X	*Eco*R I	54000 (167)	H.B. Zhang
Ae. tauschii (AL8/78)	2.2 X	*Hin*d III	59000 (189)	H.B. Zhang
*Ae. tauschii* (AL8/78)	3.2 X	*Hin*d III	52000 (190)	H.B. Zhang
*Ae. tauschii* (AL8/78)	2.8 X	*Bam*H I	59000 (149)	H.B. Zhang
*Ae. tauschii* (AL8/78)	2.4 X	*Bam*H I	76000 (174)	H.B. Zhang
*Ae. tauschii* (Aus 18913)	4.2 X	*Hin*d III	144000 (120)	E. Lagudah
*Ae. tauschii* (AS75)	4.1 X	*Bam*H I	181248 (115)	J. Dvorak
*Ae. speltoides* (2-12-4-8-1-1-1)	5.4 X	*Bam*H I	237312 (115)	J. Dvorak
*T. aestivum* (Glenlea)	3.1 X	*Bam*H I & *Hin*d III	656640 (80)	S. Cloutier
*T. aestivum* (Renan)	3.2 X	*Hin*d III	478840 (150)	B. Chalhoub
*T. aestivum* (Renan)	2.2 X	*Eco*R I	285312 (132)	B. Chalhoub
*T. aestivum* (Renan)	1.5 X	*Bam*H I	236160 (122)	B. Chalhoub
*T. aestivum* (Chinese Spring)		*Hin*d III	950000 (54)	Y. Ogihara
*T. aestivum* (Chinese Spring)	< 4%	*Mlu* I	>12000 (45)	K. Willars
		*Not* I	>1000	
*T. aestivum* (Chinese Spring) 3B	6.2 X	*Hin*d III	67968 (103)	J. Dolezel & B. Chalhoub
*T. aestivum,* (Chinese Spring) 1D, 4D & 6D	3.4 X	*Hin*d III	87168 (85)	J. Dolezel & B. Chalhoub
*T. aestivum* (Pavon) 1BS	14.5 X	*Hin*d III	65280 (82)	J. Dolezel & B. Chalhoub
*T. aestivum* (*AVS-Yr5*)	3.6 X	*Hin*d III	422400 (140)	X.M. Chen
*T. aestivum* (Norstar)	5.5 X	*Hin*d III	1200000 (75)	R. Chibbar

**Table 5:** A list of gene/QTL tagged/mapped in wheat. RSL = recombinant substitution line, CSL = chromosome substitution line, RIL = recombinant inbred lines, DH = double haploid, RICL = recombinant inbred chromosome lines, SCRI = single-chromosome recombinant lines, AL = addition lines, BIL = backcross inbred lines, NIL = near isogenic lines, TC = test cross.

Trait	Gene/QTL (chromosome)	Mapping population	Reference
Disease			
(i) Leaf rust resistance	*Lr9* (6BL)	NILs	[248]
	*Lr1* (5DL)	F_2_	[249]
	*Lr24* (3DL)	F_2_	[250]
	*Lr10* (1AS)	F_2_	[251]
	*Lr28* (4AL)	F_2:3_	[252]
	*Lr3* (6BL)	F_2_	[253]
	*Lr35* (2B)	F_2_	[254]
	*Lr47* (7A)	BC_1_F_2_	[255]
	*LrTr* (4BS)	F_2_	[256]
	*Lr19* (7DL)	Deletion lines	[257]
	*Lr39* (*=Lr41*)(2DS)	F_2_	[258]
	*Lr37* (2AS)	NILs	[259]
	*Lr20* (7AL)	F_2_	[260]
	*Lr19* (7D)	F_2_	[261]
	*Lr21/Lr40* (1DS)	F_2_	[88]
	*Lr1* (5DL)	F_2:3_ families	[262]
	*Lr28* (-)	F_2:3_	[263]
	*Lr34* (7D)	RILs	[264]
	*Lr52* (*LrW*) (5B)	F_2_	[265]
	*Lr16* (2BS)	DH	[50]
	*Lr19* (7DL)	F_2_	[266]
	*Lr24* (3DL)	F_2_	[266]
	*Lr34* (7DS)	RILs	[267]
	*Lr22a* (2DS)	F_2_	[268]
	*Lr1* (5DL)	RILs	[269]
	Unknown (5B)	F_2:3_ lines	[270]
	QTL (7D, 1BS)	RILs	[271]
	QTL (2D, 2B)	F_2_	[272]
	QTL (7DS, linked to *Lr34*)	RILs	[273]
			
(ii) Stripe rust resistance	*Yr15* (1B)	F_2_	[274]
	*YrH52* (1B)	F_2_	[275]
	*Yrns-B1* (3BS)	F_3_ lines	[276]
	*Yr15* (1B)	F_2_ lines	[277]
	*Yr28* (4DS)	RILs	[273]
	*Yr9* (1B/1R)	BC_7_F_2:3_	[278]
	*Yr17* (2A)	NILs	[259]
	*Yr26* (1BS)	F_2_ lines	[279]
	*Yr10* (1B)	F_2_ lines	[280]
	*Yr5* (2B)	BC_7_F_3_	[131]
	*Yr18* (7D)	RILs	[264]
	*Yr36* (6B)	RILs	[281]
	*YrCH42* (1B)	F_2_	[282]
	*YrZH84* (7BL)	F_2_, F_3_	[283]
	*Yr34* (5AL)	DH	[284]
	*Yr26* (1B)	F_2:3_ lines	[285]
	QTL (2D, 5B, 2B, 2A)	RILs	[286]
	QTL (2AL, 2AS, 2BL, 6BL)	DH	[287]
			
(iii) Stem rust resistance	*Sr22* (7A)	F_2_	[288]
	*Sr38* (2AS)	NILs	[259]
	*Sr2* (3BS)	F_3_ lines	[289]
			
(iv) Fusarium head blight resistance	*Fhb2* (6BS)	RILs	[290]
	QTL (5A, 3B, 1B)	DH	[37]
	QTL (3BS, 3A, 5B)	RILs	[291]
	QTL (3B)	Advanced lines	[292]
	QTL (3B, 6B, 2B)	RILs	[293, 294]
	QTL (6D, 4A, 5B)	RILs	[295]
	QTL (3A, 5A)	DH	[43]
	QTL (1B, 3B)	RILs	[30]
	QTL (2B)	RILs	[296]
	QTL (3B)	DH	[297]
	QTL (6AL, 1B, 2BL, 7BS)	RILs	[46]
	QTL (3A)	RICLs	[298]
	QTL (4D)	DH	[49]
	QTL (3BS, 5AS, 2DL)	RILs	[299]
	QTL (1BS, 1DS, 3B, 3DL, 5BL, 7BS, 7AL)	RILs	[300]
	QTL (7E)	RILs	[301]
			
(v) Scab resistance	QTL (2AS, 2BL, 3BS)	RILs	[302]
	QTL (3BS)	F_3:4_ lines	[303]
			
(vi) Powdery mildew resistance	*Pm2* (5DS)	F_2_	[304]
	*Pm18* (5DS)	F_2_	[304]
	*Pm12* (6B)	F_2_	[305]
	*Pm21* (6AL)	BC lines	[306]
	*Pm3g* (1A)	DH	[307]
	*Pm24* (1DS)	F_2:3_ lines	[308]
	*Pm26* (2BS)	RSI	[309]
	*Pm6* (2BL)	NILs	[310]
	*Pm27* (6B)	F_2_	[311]
	*Pm8/Pm17* (1BL)	F_3_ families	[312]
	*Pm3* (1AS)	RILs	[313]
	*Pm1* (7AL)	F_2_	[260]
	*Pm29* (7D)	F_2_ * *&* *F_4_ lines	[314]
	*Pm30* (5BS)	BC_2_F_2_ lines	[315]
	*Pm13* (3S)	AL	[316]
	*Pm5e* (7BL)	F_2_	[130]
	*Pm4a* (2A)	F_2_	[317]
	*PmU* (7AL)	F_2_	[318]
	*Pm34* (5D)	F_2:3_ lines	[319]
	*PmY39* (2B)	BC_3_F_4:5_	[320]
	*Pm35* (5DL)	F_2:3_ lines	[321]
	*Pm5d* (7BL)	F_3_ lines	[322]
	*Pm12* (6B)	BC_3_F_2_	[323]
	*MlRE*, QTL (6A, 5D)	F_3_ lines	[324]
	*MlG* (6AL)	BC_2_F_3_	[325]
	*mlRD30* (7AL)	F_2_	[326]
	*Mlm2033, Mlm80* (7A)	F_2_	[181]
	QTL (5A, 7B, 3D)	RILs	[327]
	QTL (1B, 2A, 2B)	F_2:3_ lines	[328]
	QTL (2B, 5D, 6A)	DH	[329]
	QTL (2B)	F_2_	[330]
	QTL (1BL, 2AL, 2BL)	RILs	[331]
			
(vii) Common bunt resistance	*Bt-10* (-)	F_2_	[332]
	QTL (1B, 7A)	DH	[333]
(viii) Tan spot and *Stagonospora nodorum* blotch resistance	QTL (1A, 4A, 1B, 3B)	RILs	[334]
	QTL (5B, 3B)	Inbred, CS lines	[335]
	*tsn3a*, *tsn3b*, *tsn3c* (3D)	F_2:3_ lines	[336]
(ix) Septoria tritici blotch resistance	*Stb5* (7D)	SCRI	[337]
	QTL (3A)	DH	[38]
	QTL (1D, 2D, 6B)	RILs	[338]
(x) Barley yellow dwarf tolerance	QTL (12 chromosomes)	RILs	[339]
(xi) Leaf and glume blotch resistance	QTL (4B, 7B, 5A)	RILs	[340]
(xii) Wheat streak mosaic virus resistance	*Wms1* (4D)	F_2_	[341]
	*WSSMV* (2DL)	RILs	[342]
(xiii) Yellow mosaic virus resistance	*YmYF* (2D)	F_2_	[343]
(xiv) Eyespot (straw breaker foot rot) resistance	*Pch2* (7AL)	F_2_	[344]
	*Pch1* (7A)	F_3_ lines	[345]
	*Pch1, Ep-D1* (7D)	TC	[346]

Insect-pest			
(i) Green bug resistance	*Gb3* (7D)	F_2:3_ lines	[347]
	*Gby* (7A)	F_2:3_ lines	[348]
	*Gb7* (7DL)	RILs	[349]
	*Gb* (7DL)	F_4:5_ lines, F_2_	[350]
			
(ii) Hessian fly resistance gene	*H23* (6D)	F_2_	[351]
	*H24* (3D)	F_2_	[351]
	*H3, H6, H9, H10, H12, H16, H17* (5A)	NILs, F_2_	[352]
	*H5, H11, H13, H14* (1A)	NILs, F_2_	[353]
	*H21* (2B)	NILs, F_2_	[354]
	*H6* (-)	F_2_	[355]
	*H13* (6DS)	F_2:3_	[356]
	*H26, H13* (3D, 6D)	F_2:3_ lines	[357]
	*H22* (1D)	F_2:3_ lines	[358]
	*H16 and H17* (1AS)	BC_1_F_2_, F_2:3_ lines	[359]
			
(iii) Russian wheat aphid resistance	*Dn8, Dn9* (7DS, 1DL)	F_2_	[360]
	*Dn1, Dn2, Dn5, Dn8*	F_2_	[360]
	*Dnx* (7DS)		
	*Dn2* (7DS)	F_2_	[361]
	*Dn4* (1D)	F_2_	[362]
	*Dn6* (7D)	F_2_	[362]

Nematodes			
(i) Cereal cyst nematode resistance	*Cre1* (2B)	NILs, F_2_	[363]
	*Cre5* (2AS)	NILs	[364]
	*Cre6* (5A)	F_2_	[365]
	QTL (1B)	DH	[48]
(ii) Root-knot nematode resistance	*Rkn-mn1* (3BL)	BC_3_F_2_, F_3_ lines	[366]
(iii) Root-lesion nematode resistance	*Rlnn1* (7AL)	DH	[367]

Quality and quality related traits			
(i) Seed dormancy or preharvest sprouting	QTL (4A)	DH	[368]
	QTL (4A)	RILs, DH	[369]
	QTL (3A)	BC_1_F_2_	[370]
	QTL (3A)	RILs	[371]
	QTL (3A)	RILs	[372]
	QTL (4A)	DH	[373]
			
(ii) Grain protein content	QTL (6B)	RILs	[374]
	QTL (2A, 3A, 4D, 7D, 2B, 5B, 7A)	RILs	[39]
	QTL (2A, 2B, 2D, 3D, 4A, 6B, 7A, 7D)	RILs	[375]
	QTL (2AS, 6AS, 7BL)	BILs	[376]
			
(iii) Others			
Flour colour	QTL (3A, 7A)	RILs	[377]
Milling yield	QTL (3A, 7D)	RILs	[378]
Bread-making quality	QTL (5DS, 1B, 6A, 3B, 1A)	DH	[379]
Milling traits	QTL (7A, 6B)	RILs	[35]
Grain dry matter and N accumulation, protein composition	QTL (1A, 2B, 3A, 6A, 5A, 7A, 7D)	RILs	[380]
Mixograph-extensibility	QTL (5A)	DH	[381]
Kernel hardness and dough strength	QTL (1A, 5D, 1B, 1D, 5B)	Inbred lines	[382]
Purple grain colour	*Pp1, Pp3b, Pp3a* (2A, 7BL)	F_2_	[383]
Quality traits	QTL (5DS, 6DS, 2DS, 1AS, 1BS, 6DS)	RILs	[384]
Low-molecular-weight glutenin	*LMW-GS* (-)	F_5_ lines	[385]
Bread-making quality	QTL (3A, 7A)	RILs	[40]
Milling and baking quality	QTL (4B, 6D)	BC_2_F_3_	[230]
Endosperm colour	QTL, *Psy1-1* (2A, 4B, 6B, 7B)	DH	[386]

Agronomic traits			
(i) Plant height	*Rht-B1*, *Rht-D1* (4BS, 4DS)	DH	[387]
	*Rht8* (2D)	RILs	[388]
	*Rht8* (2DS)	DH, Inbred lines	[389]
(ii) Tiller inhibition gene	*tin3* (3A)	F_2_	[390]
(iii) Spherical grain and compact spikes	*s16219, C17648* *B1* (3B, 5A)	F_2_	[391]
(iv) Ear-emergence time and plant height	QTL (5A)	RILs	[392]
			
(v) Heading date	QTL (2BS)	DH	[393]
	QTL, *Ppd-B1,* *Ppd-D1* (2B, 2D, 5A, 2B)	RILs	[394]
	QTL (2DS)	RILs	[395]
	QTL (2A, 2B, 2D, 5A, 5B, 5D, 4A, 4B)	RILs	[396]
			
(vi) Grain yield and related traits	QTL (5A)	RILs	[397]
	QTL (2D, 3B, 3D, 5D, 7D)	BC_2_F_2:4_ lines	[398]
	QTL (1D, 2A, 6B, 7D)	RILs	[51]
	QTL (7AL, 7BL, 1D, 5A)	DH	[45]
	QTL (4AL)	RILs	[399]
	QTL (1B, 4D, 7D)	DH	[400]
(vii) Spike-related traits	QTL (7D)	F_2_	[401]
			
(viii) Grain weight	QTL (1A, 2B, 7A)	RILs	[402]
(ix) Others	QTL (4A, 4B, 4D, 7D, 3B, 3D)	DH	[171]
	QTL (1D, 4D)	DH	[47]

Growth related traits			
(i) Spike morphology, awn development, vernalization	*B, Q, VRN1* (5A)	RILs	[403]
(ii) Supernumerary spikelet	*bh* (2D, 4A, 4B, 5A)	F_2:3_ lines	[404]
(iii) Sphaerococcum- like growth habit	*S1, S2, S3* (3D, 3B, 3A)	F_2_	[405]
(iv) Thermosensitive earliness	*Eps-Am1* (1AL)	F_2_	[406]
(v) Coleoptiles pigmentation	*Rc-A1, Rc-B1*, *Rc-D1* (7A, 7B, 7D, 4BL)	RILs	[407]
(vi) Thermosensitive genic male-sterile	*wtms1* (2B)	F_2_	[408]
(vii) Hybrid necrosis	*Ne1, Ne2* (5BL, 2BS)	F_2_	[409]
(viii) Leaf pubescence and hairy leaf	*Hl1, Hl2, Aesp*, QTL (4BL, 7BS)	F_2_	[410]
(ix) Stem solidness	*sst* (3BL)	DH	[411]
(x) Lodging resistance	QTL (1BS, 2AS, 2D, 3AS, 4AS, 5AL, 5BL, 6BL, 7BL)	RILs	[412]
(xi) Stem strength and related traits	QTL (3A, 3B, 1A, 2D)	DH	[413]
(xii) Brittle rachis	QTL (3A, 3B)	RICLs	[414]
(xiii) Coleoptiles growth	QTL (2B, 2D, 4A, 5D, 6B)	DH	[415]
(xiv) Kernel shattering	QTL (2B, 3B, 7A)	RILs	[416]
(xv) Seed development	QTL (1D, 4B)	DH	[9]
(xvi) Longer coleoptiles	QTL (6A)	RILs	[29]
(xvii) Viridescent phenotype	QTL (2B)	DH	[52]

Biochemical			
(i) Casein kinase	*CK2*α** (5A)	F_2_	[417]
(ii) Nonglaucousness	*Iw3672* (2DS)	F_2_	[418]
(iii) Low lipoxygenase	*Lpx-B1.1, Lpx-A3* (4B, 4A)	RILs	[419]
(iv) Polyphenol oxidase (PPO) genes	*PPO* (2A, 2D)	DH	[420]
(v) ABA signaling genes	QTL (3A, 5A)	RILs	[421]
(vi) Polyphenol oxidase	QTL (2A)	DH	[422]
(vii) Water-soluble carbohydrates	QTL (21 chromosome)	DH	[423]

Abiotic stress			
(i) Photoperiod insensitive	*Ppd-B1* (2BS)	RILs, DH	[424]
(ii) Aluminum tolerance	*ALMT1* (4D)	DH	[425]
	QTL (4D, 3BL)	RIL	[426]
(iii) Boron toxicity tolerance	QTL (*Bo1*) (7BL)	DH	[427]
(iv) Frost resistance	QTL (5B)	RSI	[428]
(v) Salt tolerance	QTL (3A, 3B, 4DL, 6DL)	RILs	[429]

## References

[1] Gill BS, Appels R, Botha-Oberholster A-M (2004). A workshop report on wheat genome sequencing: international genome research on wheat consortium. *Genetics*.

[2] Sears ER, Riley R, Lewis KR (1966). Nullisomic-tetrasomic combinations in hexaploid wheat. *Chromosome Manipulation and Plant Genetics*.

[3] Endo TR, Gill BS (1996). The deletion stocks of common wheat. *Journal of Heredity*.

[4] Gill KS, Gupta PK, Varshney RK (2004). Gene distribution in cereal genomes. *Cereal Genomics*.

[5] Singh K, Ghai M, Garg M (2007). An integrated molecular linkage map of diploid wheat based on a *Triticum boeoticum × T. monococcum* RIL population. *Theoretical and Applied Genetics*.

[6] Feuillet C, Keller B (2002). Comparative genomics in the grass family: molecular characterization of grass genome structure and evolution. *Annals of Botany*.

[7] Gale MD, Devos KM (1998). Plant comparative genetics after 10 years. *Science*.

[8] Devos KM (2005). Updating the ‘crop circle’. *Current Opinion in Plant Biology*.

[9] Jordan MC, Somers DJ, Banks TW (2007). Identifying regions of the wheat genome controlling seed development by mapping expression quantitative trait loci. *Plant Biotechnology Journal*.

[10] Bagge M, Xia X, Lübberstedt T (2007). Functional markers in wheat. *Current Opinion in Plant Biology*.

[11] Moolhuijzen P, Dunn DS, Bellgard M (2007). Wheat genome structure and function: genome sequence data and the international wheat genome sequencing consortium. *Australian Journal of Agricultural Research*.

[12] Somers DJ, Tsunewaki K (2005). Molecular breeding and assembly of complex genotypes in wheat. *Frontiers of Wheat Bioscience. The 100 Memorial Issue of Wheat Information Service*.

[13] Chao S, Sharp PJ, Worland AJ, Warham EJ, Koebner RMD, Gale MD (1989). RFLP-based genetic maps of wheat homoeologous group 7 chromosomes. *Theoretical and Applied Genetics*.

[14] Gupta PK, Varshney RK, Sharma PC, Ramesh B (1999). Molecular markers and their applications in wheat breeding. *Plant Breeding*.

[15] Appels R, Pogna NE A consensus molecular genetic map of wheat-a cooperative international effort.

[16] Somers DJ, Isaac P, Edwards K (2004). A high-density microsatellite consensus map for bread wheat (*Triticum aestivum* L.). *Theoretical and Applied Genetics*.

[17] McIntosh RA, Devos KM, Dubcovsky J, Morris CF, Rogers WJ Catalogue of gene symbols for wheat. http://wheat.pw.usda.gov/ggpages/wgc/2003upd.html.

[18] Röder MS, Korzun V, Wendehake K (1998). A microsatellite map of wheat. *Genetics*.

[19] Pestsova E, Ganal MW, Röder MS (2000). Isolation and mapping of microsatellite markers specific for the D genome of bread wheat. *Genome*.

[20] Gupta PK, Balyan HS, Edwards KJ (2002). Genetic mapping of 66 new microsatellite (SSR) loci in bread wheat. *Theoretical and Applied Genetics*.

[21] Gao LF, Jing RL, Huo NX (2004). One hundred and one new microsatellite loci derived from ESTs (EST-SSRs) in bread wheat. *Theoretical and Applied Genetics*.

[22] Yu J-K, Dake TM, Singh S (2004). Development and mapping of EST-derived simple sequence repeat markers for hexaploid wheat. *Genome*.

[23] Nicot N, Chiquet V, Gandon B (2004). Study of simple sequence repeat (SSR) markers from wheat expressed sequence tags (ESTs). *Theoretical and Applied Genetics*.

[24] Snape JW, Moore G, Buck HT (2007). Reflections and opportunities: gene discovery in the complex wheat genome. *Wheat Production in Stressed Environments*.

[25] Gill KS, Lubbers EL, Gill BS, Raupp WJ, Cox TS (1991). A genetic linkage map of *Triticum tauschii* (DD) and its relationship to the D genome of bread wheat (AABBDD). *Genome*.

[26] Boyko EV, Gill BS, Mickelson-Young L (1999). A high-density genetic linkage map of *Aegilops tauschii*, the D-genome progenitor of bread wheat. *Theoretical and Applied Genetics*.

[27] Song QJ, Shi JR, Singh S (2005). Development and mapping of microsatellite (SSR) markers in wheat. *Theoretical and Applied Genetics*.

[28] Torada A, Koike M, Mochida K, Ogihara Y (2006). SSR-based linkage map with new markers using an intraspecific population of common wheat. *Theoretical and Applied Genetics*.

[29] Spielmeyer W, Hyles J, Joaquim P (2007). A QTL on chromosome 6A in bread wheat (*Triticum aestivum*) is associated with longer coleoptiles, greater seedling vigour and final plant height. *Theoretical and Applied Genetics*.

[30] Zhang X, Zhou M, Ren L (2004). Molecular characterization of *Fusarium* head blight resistance from wheat variety Wangshuibai. *Euphytica*.

[31] Dubcovsky J, Luo M-C, Zhong G-Y (1996). Genetic map of diploid wheat, *Triticum monococcum* L., and its comparison with maps of *Hordeum vulgare* L.. *Genetics*.

[32] Blanco A, Bellomo MP, Cenci A (1998). A genetic linkage map of durum wheat. *Theoretical and Applied Genetics*.

[33] Lotti C, Salvi S, Pasqualone A, Tuberosa R, Blanco A (2000). Integration of AFLP markers into an RFLP-based map of durum wheat. *Plant Breeding*.

[34] Nachit MM, Elouafi I, Pagnotta MA (2001). Molecular linkage map for an intraspecific recombinant inbred population of durum wheat (*Triticum turgidum* L. var. *durum *). *Theoretical and Applied Genetics*.

[35] Elouafi I, Nachit MM (2004). A genetic linkage map of the Durum × *Triticum dicoccoides* backcross population based on SSRs and AFLP markers, and QTL analysis for milling traits. *Theoretical and Applied Genetics*.

[36] Messmer MM, Keller M, Zanetti S, Keller B (1999). Genetic linkage map of a wheat × spelt cross. *Theoretical and Applied Genetics*.

[37] Buerstmayr H, Lemmens M, Hartl L (2002). Molecular mapping of QTLs for Fusarium head blight resistance in spring wheat. I. Resistance to fungal spread (type II resistance). *Theoretical and Applied Genetics*.

[38] Eriksen L, Borum F, Jahoor A (2003). Inheritance and localisation of resistance to *Mycosphaerella graminicola* causing septoria tritici blotch and plant height in the wheat (*Triticum aestivum* L.) genome with DNA markers. *Theoretical and Applied Genetics*.

[39] Groos C, Robert N, Bervas E, Charmet G (2003). Genetic analysis of grain protein-content, grain yield and thousand-kernel weight in bread wheat. *Theoretical and Applied Genetics*.

[40] Groos C, Bervas E, Chanliaud E, Charmet G (2007). Genetic analysis of bread-making quality scores in bread wheat using a recombinant inbred line population. *Theoretical and Applied Genetics*.

[41] Paillard S, Schnurbusch T, Winzeler M (2003). An integrative genetic linkage map of winter wheat (*Triticum aestivum* L.). *Theoretical and Applied Genetics*.

[42] Sourdille P, Cadalen T, Guyomarc'h H (2003). An update of the Courtot × Chinese Spring intervarietal molecular marker linkage map for the QTL detection of agronomic traits in wheat. *Theoretical and Applied Genetics*.

[43] Steiner B, Lemmens M, Griesser M, Scholz U, Schondelmaier J, Buerstmayr H (2004). Molecular mapping of resistance to *Fusarium* head blight in the spring wheat cultivar Frontana. *Theoretical and Applied Genetics*.

[44] Liu ZH, Anderson JA, Hu J, Friesen TL, Rasmussen JB, Faris JD (2005). A wheat intervarietal genetic linkage map based on microsatellite and target region amplified polymorphism markers and its utility for detecting quantitative trait loci. *Theoretical and Applied Genetics*.

[45] Quarrie SA, Steed A, Calestani C (2005). A high-density genetic map of hexaploid wheat (*Triticum aestivum* L.) from the cross Chinese Spring × SQ1 and its use to compare QTLs for grain yield across a range of environments. *Theoretical and Applied Genetics*.

[46] Schmolke M, Zimmermann G, Buerstmayr H (2005). Molecular mapping of Fusarium head blight resistance in the winter wheat population Dream/Lynx. *Theoretical and Applied Genetics*.

[47] Huang XQ, Cloutier S, Lycar L (2006). Molecular detection of QTLs for agronomic and quality traits in a doubled haploid population derived from two Canadian wheats (*Triticum aestivum* L.). *Theoretical and Applied Genetics*.

[48] Williams KJ, Willsmore KL, Olson S, Matic M, Kuchel H (2006). Mapping of a novel QTL for resistance to cereal cyst nematode in wheat. *Theoretical and Applied Genetics*.

[49] Draeger R, Gosman N, Steed A (2007). Identification of QTLs for resistance to Fusarium head blight, DON accumulation and associated traits in the winter wheat variety Arina. *Theoretical and Applied Genetics*.

[50] McCartney CA, Somers DJ, McCallum BD (2005). Microsatellite tagging of the leaf rust resistance gene *Lr16* on wheat chromosome 2BSc. *Molecular Breeding*.

[51] Li S, Jia J, Wei X (2007). A intervarietal genetic map and QTL analysis for yield traits in wheat. *Molecular Breeding*.

[52] Simmonds JR, Fish LJ, Leverington-Waite MA, Wang Y, Howell P, Snape JW (2008). Mapping of a gene (*Vir*) for a non-glaucous, viridescent phenotype in bread wheat derived from *Triticum dicoccoides*, and its association with yield variation. *Euphytica*.

[53] Akbari M, Wenzl P, Caig V (2006). Diversity arrays technology (DArT) for high-throughput profiling of the hexaploid wheat genome. *Theoretical and Applied Genetics*.

[54] Semagn K, Bjørnstad Å, Skinnes H, Marøy AG, Tarkegne Y, William M (2006). Distribution of DArT, AFLP, and SSR markers in a genetic linkage map of a doubled-haploid hexaploid wheat population. *Genome*.

[55] Sears ER (1954). The aneuploids of common wheat. *University of Missouri Agriculture Experiment Station, Bulleten*.

[56] Qi LL, Echalier B, Chao S (2004). A chromosome bin map of 16,000 expressed sequence tag loci and distribution of genes among the three genomes of polyploid wheat. *Genetics*.

[57] Kota RS, Gill KS, Gill BS, Endo TR (1993). A cytogenetically based physical map of chromosome 1B in common wheat. *Genome*.

[58] Gill KS, Gill BS, Endo TR, Taylor T (1996). Identification and high-density mapping of gene-rich regions in chromosome group 1 of wheat. *Genetics*.

[59] Delaney DE, Nasuda S, Endo TR, Gill BS, Hulbert SH (1995). Cytologically based physical maps of the group-2 chromosomes of wheat. *Theoretical and Applied Genetics*.

[60] Röder MS, Korzun V, Gill BS, Ganal MW (1998). The physical mapping of microsatellite markers in wheat. *Genome*.

[61] Delaney DE, Nasuda S, Endo TR, Gill BS, Hulbert SH (1995). Cytologically based physical maps of the group 3 chromosomes of wheat. *Theoretical and Applied Genetics*.

[62] Mickelson-Young L, Endo TR, Gill BS (1995). A cytogenetic ladder-map of the wheat homoeologous group-4 chromosomes. *Theoretical and Applied Genetics*.

[63] Gill KS, Gill BS, Endo TR, Boyko EV (1996). Identification and high-density mapping of gene-rich regions in chromosome group 5 of wheat. *Genetics*.

[64] Faris JD, Haen KM, Gill BS (2000). Saturation mapping of a gene-rich recombination hot spot region in wheat. *Genetics*.

[65] Qi LL, Gill BS (2001). High-density physical maps reveal that the dominant male-sterile gene Ms3 is located in a genomic region of low recombination in wheat and is not amenable to map-based cloning. *Theoretical and Applied Genetics*.

[66] Ogihara Y, Hasegawa K, Tsujimoto H (1994). High-resolution cytological mapping of the long arm of chromosome 5A in common wheat using a series of deletion lines induced by gametocidal (Gc) genes of *Aegilops speltoides*. *Molecular and General Genetics*.

[67] Gill KS, Gill BS, Endo TR (1993). A chromosome region-specific mapping strategy reveals gene-rich telomeric ends in wheat. *Chromosoma*.

[68] Weng Y, Tuleen NA, Hart GE (2000). Extended physical maps and a consensus physical map of the homoeologous group-6 chromosomes of wheat (*Triticum aestivum* L. em Thell.). *Theoretical and Applied Genetics*.

[69] Weng Y, Lazar MD (2002). Comparison of homoeologous group-6 short arm physical maps of wheat and barley reveals a similar distribution of recombinogenic and gene-rich regions. *Theoretical and Applied Genetics*.

[70] Werner JE, Endo TR, Gill BS (1992). Towards a cytogenetically based physical map of the wheat genome. *Proceedings of the National Academy of Sciences of the United States of America*.

[71] Hohmann U, Endo TR, Gill KS, Gill BS (1994). Comparison of genetic and physical maps of group 7 chromosomes from *Triticum aestivum* L. *Molecular and General Genetics*.

[72] Varshney RK, Prasad M, Roy JK, Röder MS, Balyan HS, Gupta PK (2001). Integrated physical maps of 2DL, 6BS and 7DL carrying loci for grain protein content and pre-harvest sprouting tolerance in bread wheat. *Cereal Research Communications*.

[73] Zhang H, Nasuda S, Endo TR (2000). Identification of AFLP markers on the satellite region of chromosome 1BS in wheat. *Genome*.

[74] Rodriguez Milla MA, Gustafson JP (2001). Genetic and physical characterization of chromosome 4DL in wheat. *Genome*.

[75] Sandhu D, Sidhu D, Gill KS (2002). Identification of expressed sequence markers for a major gene-rich region of 
wheat chromosome group 1 using RNA fingerprinting-differential display. *Crop Science*.

[76] Sourdille P, Singh S, Cadalen T (2004). Microsatellite-based deletion bin system for the establishment of genetic-physical map relationships in wheat (*Triticum aestivum* L.). *Functional and Integrative Genomics*.

[77] Goyal A, Bandopadhyay R, Sourdille P, Endo TR, Balyan HS, Gupta PK (2005). Physical molecular maps of wheat chromosomes. *Functional & Integrative Genomics*.

[78] Peng JH, Lapitan NLV (2005). Characterization of EST-derived microsatellites in the wheat genome and development of eSSR markers. *Functional and Integrative Genomics*.

[79] Mohammedan A, Goyal A, Singh R, Balyan HS, Gupta PK (2007). Physical mapping of wheat and rye expressed sequence tag-simple 
sequence repeats on wheat chromosomes. *Crop Science*.

[80] Parida SK, Raj Kumar KA, Dalal V, Singh NK, Mohammedapatra T (2006). Unigene derived microsatellite markers for the cereal genomes. *Theoretical and Applied Genetics*.

[81] Hill-Ambroz K, Webb CA, Matthews AR, Li W, Gill BS, Fellers JP (2006). Expression analysis and physical mapping of a cDNA library of Fusarium head blight infected wheat spikes. *Crop Science*.

[82] Goss SJ, Harris H (1975). New method for mapping genes in human chromosomes. *Nature*.

[83] Cox DR, Burmeister M, Price ER, Kim S, Myers RM (1990). Radiation hybrid mapping: a somatic cell genetic method for constructing high-resolution maps of mammalian chromosomes. *Science*.

[84] Kalavacharla V, Hossain K, Gu Y (2006). High-resolution radiation hybrid map of wheat chromosome 1D. *Genetics*.

[85] Ling H-Q, Zhu Y, Keller B (2003). High-resolution mapping of the leaf rust disease resistance gene *L*
*r*1 in wheat and characterization of BAC clones from the *L*
*r*1 locus. *Theoretical and Applied Genetics*.

[86] Cloutier S, McCallum BD, Loutre C (2007). Leaf rust resistance gene *Lr1*, isolated from bread wheat (*Triticum aestivum* L.) is a member of the large *psr567* gene family. *Plant Molecular Biology*.

[87] Feuillet C, Travella S, Stein N, Albar L, Nublat A, Keller B (2003). Map-based isolation of the leaf rust disease resistance gene *Lr10* from the hexaploid wheat (*Triticum aestivum* L.) genome. *Proceedings of the National Academy of Sciences of the United States of America*.

[88] Huang L, Brooks SA, Li W, Fellers JP, Trick HN, Gill BS (2003). Map-based cloning of leaf rust resistance gene *L*
*r*21 from the large and polyploid genome of bread wheat. *Genetics*.

[89] Yan L, Loukoianov A, Tranquilli G, Helguera M, Fahima T, Dubcovsky J (2003). Positional cloning of the wheat vernalization gene *VRN1*. *Proceedings of the National Academy of Sciences of the United States of America*.

[90] Yan L, Loukoianov A, Blechl A (2004). The wheat *VRN2* gene is a flowering repressor down-regulated by vernalization. *Science*.

[91] Yan L, Fu D, Li C (2006). The wheat and barley vernalization gene *VRN3* is an orthologue of FT. *Proceedings of the National Academy of Sciences of the United States of America*.

[92] Simons KJ, Fellers JP, Trick HN (2006). Molecular characterization of the major wheat domestication gene Q. *Genetics*.

[93] Faris JD, Fellers JP, Brooks SA, Gill BS (2003). A bacterial artificial chromosome contig spanning the major domestication locus *Q* in wheat and identification of a candidate gene. *Genetics*.

[94] Yahiaoui N, Srichumpa P, Dudler R, Keller B (2004). Genome analysis at different ploidy levels allows cloning of the powdery mildew resistance gene *Pm3b* from hexaploid wheat. *Plant Journal*.

[95] Brunner S, Srichumpa P, Yahiaoui N, Keller B Positional cloning and evolution of powdery mildew resistance gene at *Pm3* locus of hexaploid wheat.

[96] Uauy C, Distelfeld A, Fahima T, Blechl A, Dubcovsky J (2006). A NAC gene regulating senescence improves grain protein, zinc, and iron content in wheat. *Science*.

[97] Distelfeld A, Uauy C, Olmos S, Schlatter AR, Dubcovsky J, Fahima T (2004). Microcolinearity between a 2-cM region encompassing the grain protein content locus *Gpc-6B1* on wheat chromosome 6B and a 350-kb region on rice chromosome 2. *Functional & Integrative Genomics*.

[98] Liu S, Pumphery MO, Zhang X Towards positional cloning of Qfhs.ndsu-3BS, a major QTL for Fusarium head blight resistance in wheat.

[99] Ling P, Chen X, Le DQ, Campbell KG Towards cloning of the *Y*
*r*5 gene for resistance to wheat stripe rust resistance.

[100] Schnurbusch T, Collins NC, Eastwood RF, Sutton T, Jefferies SP, Langridge P (2007). Fine mapping and targeted SNP survey using rice-wheat gene colinearity in the region of the 
*Bo1* boron toxicity tolerance locus of bread wheat. *Theoretical and Applied Genetics*.

[101] Lu H-J, Fellers JP, Friesen TL, Meinhardt SW, Faris JD (2006). Genomic analysis and marker development for the *Tsn1* locus in wheat using bin-mapped ESTs and flanking BAC contigs. *Theoretical and Applied Genetics*.

[102] Griffiths S, Sharp R, Foote TN (2006). Molecular characterization of *P*
*h*1 as a major chromosome pairing locus in polyploid wheat. *Nature*.

[103] Kota RS, Spielmeyer W, McIntosh RA, Lagudah ES (2006). Fine genetic mapping fails to dissociate durable stem rust resistance gene *S*
*r*2 from pseudo-black chaff in common wheat (*Triticum aestivum* L.). *Theoretical and Applied Genetics*.

[104] Flavell RB, Smith DB (1974). The role of homoeologous group 1 chromosomes in the control of rRNA genes in wheat. *Biochemical Genetics*.

[105] Gerlach WL, Peacock WJ (1980). Chromosomal locations of highly repeated DNA sequences in wheat. *Heredity*.

[106] Gerlach WL, Dennis ES, Peacock WJ, Swaminathan MS, Gupta PK, Sinha U (1983). Molecular cytogenetics of wheat. *Cytogenetics of Crop Plant*.

[107] Mukai Y, Nakahara Y, Yamamoto M (1993). Simultaneous discrimination of the three genomes in hexaploid wheat by multicolor fluorescence 
*in situ* hybridization using total genomic and highly repeated DNA probes. *Genome*.

[108] Pedersen C, Langridge P (1997). Identification of the entire chromosome complement of bread wheat by two-colour FISH. *Genome*.

[109] Badaeva ED, Amosova AV, Muravenko OV (2002). Genome differentiation in *Aegilops*. 3. Evolution of the D-genome cluster. *Plant Systematics and Evolution*.

[110] Zhang P, Li W, Friebe B, Gill BS (2004). Simultaneous painting of three genomes in hexaploid wheat by BAC-FISH. *Genome*.

[111] Mukai Y, Endo TR, Gill BS (1990). Physical mapping of the 5S rRNA multigene family in common wheat. *Journal of Heredity*.

[112] Mukai Y, Endo TR, Gill BS (1991). Pysical mapping of the 18S.26S rRNA multigene family in common wheat: identification of a new locus. *Chromosoma*.

[113] Ma X-F, Ross K, Gustafson JP (2001). Physical mapping of restriction fragment length polymorphism (RFLP) markers in homoeologous groups 1 and 3 chromosomes of wheat by *in situ* hybridization. *Genome*.

[114] Rahman S, Regina A, Li Z (2001). Comparison of starch-branching enzyme genes reveals evolutionary relationships among isoforms. Characterization of a gene for starch-branching enzyme IIa from the wheat D genome donor 
*Aegilops tauschii*. *Plant Physiology*.

[115] Li Z, Sun F, Xu S (2003). The structural organisation of the genes encoding class II starch synthase of wheat and barley and the evolution of the genes encoding starch syuthases in plants. *Functional & Integrative Genomics*.

[116] Turnbull K-M, Turner M, Mukai Y (2003). The organization of genes tightly linked to the Ha locus in *Aegilops tauschii*, the D-genome donor to wheat. *Genome*.

[117] Mukai Y, Gill BS (1991). Detection of barley chromatin added to wheat by genomic *in situ* hybridization. *Genome*.

[118] Schwarzacher T, Anamthawat-Jónsson K, Harrison GE (1992). Genomic *in situ* hybridization to identify alien chromosomes and chromosome segments 
in wheat. *Theoretical and Applied Genetics*.

[119] Biagetti M, Vitellozzi F, Ceoloni C (1999). Physical mapping of wheat-*Aegilops longissima* breakpoints in mildew-resistant recombinant lines using FISH with highly repeated and low-copy DNA probes. *Genome*.

[120] Yamamoto M, Mukai Y, Slinkard AE High-resolution mapping in wheat and rye by FISH on extended DNA fibres.

[121] Yamamoto M, Mukai Y (2005). High-resolution physical mapping of the secalin-1 locus of rye on extended DNA fibers. *Cytogenetic and Genome Research*.

[122] Lavania UC, Yamamoto M, Mukai Y (2003). Extended chromatin and DNA fibers from active plant nuclei for high-resolution FISH. *Journal of Histochemistry & Cytochemistry*.

[123] Fukui K-N, Suzuki G, Lagudah ES (2001). Physical arrangement of retrotransposon-related repeats in centromeric regions of wheat. *Plant & Cell Physiology*.

[124] Valárik M, Bartoš J, Kovářová P, Kubaláková M, de Jong JH, Doležel J (2004). High-resolution FISH on super-stretched flow-sorted plant chromosomes. *Plant Journal*.

[125] Jackson SA, Zhang P, Chen WP (2001). High-resolution structural analysis of biolistic transgene integration into the genome of wheat. *Theoretical and Applied Genetics*.

[126] Zhang P, Friebe B, Gill B Potential and limitations of BAC-FISH mapping in wheat.

[127] Papa D, Miller CA, Anderson GR FISH physical mapping of DNA sequences associated with RWA resistance in wheat and barley.

[128] Zhang P, Li W, Fellers J, Friebe B, Gill BS (2004). BAC-FISH in wheat identifies chromosome landmarks consisting of different types of transposable elements. *Chromosoma*.

[129] Huang XQ, Cöster H, Ganal MW, Röder MS (2003). Advanced backcross QTL analysis for the identification of quantitative trait loci alleles from wild relatives of wheat (*Triticum aestivum* L.). *Theoretical and Applied Genetics*.

[130] Huang XQ, Wang LX, Xu MX, Röder MS (2003). Microsatellite mapping of the powdery mildew resistance gene *P*
*m*5*e* in common wheat (*Triticum aestivum* L.). *Theoretical and Applied Genetics*.

[131] Yan GP, Chen XM, Line RF, Wellings CR (2003). Resistance gene-analog polymorphism markers co-segregating with the *Yr5* gene for resistance to wheat stripe rust. *Theoretical and Applied Genetics*.

[132] Gupta PK, Rustgi S, Sharma S, Singh R, Kumar N, Balyan HS (2003). Transferable EST-SSR markers for the study of polymorphism and genetic diversity in bread wheat. *Molecular Genetics and Genomics*.

[133] Gao LF, Tang J, Li H, Jia J (2003). Analysis of microsatellites in major crops assessed by computational and experimental approaches. *Molecular Breeding*.

[134] Bandopadhyay R, Sharma S, Rustgi S (2004). DNA polymorphism among 18 species of *Triticum-Aegilops* complex using wheat EST-SSRs. *Plant Science*.

[135] Varshney RK, Sigmund R, Börner A (2005). Interspecific transferability and comparative mapping of barley EST-SSR markers in wheat, rye and rice. *Plant Science*.

[136] Yu J-K, La Rota M, Kantety RV, Sorrells ME (2004). EST derived SSR markers for comparative mapping in wheat and rice. *Molecular Genetics and Genomics*.

[137] Zhang LY, Bernard M, Leroy P, Feuillet C, Sourdille P (2005). High transferability of bread wheat EST-derived SSRs to other cereals. *Theoretical and Applied Genetics*.

[138] Tang J, Gao L, Cao Y, Jia J (2006). Homologous analysis of SSR-ESTs and transferability of wheat SSR-EST markers across barley, rice and maize. *Euphytica*.

[139] Chabane K, Abdalla O, Sayed H, Valkoun J (2007). Assessment of EST-microsatellites markers for discrimination and genetic diversity in bread and durum wheat landraces from Afghanistan. *Genetic Resources and Crop Evolution*.

[140] Zhang W, Chao S, Akhunov ED Discovery of SNPs for wheat homoeologous group 5 and polymorphism among US adapted wheat germplasm.

[141] Ravel C, Praud S, Murigneux A (2006). Single-nucleotide polymorphism frequency in a set of selected lines of bread wheat (*Triticum aestivum* L.). *Genome*.

[142] Janda J, Bartoš J, Šafář J (2004). Construction of a subgenomic BAC library specific for chromosomes 1D, 4D and 6D of hexaploid wheat. *Theoretical and Applied Genetics*.

[143] Šafář J, Bartoš J, Janda J (2004). Dissecting large and complex genomes: flow sorting and BAC cloning of individual 
chromosomes from bread wheat. *The Plant Journal*.

[144] Janda J, Šafář J, Kubaláková M (2006). Advanced resources for plant genomics: a BAC library specific for the short arm of wheat chromosome 1B. *The Plant Journal*.

[145] Wicker T, Stein N, Albar L, Feuillet C, Schlagenhauf E, Keller B (2001). Analysis of a contiguous 211 kb sequence in diploid wheat (*Triticum monococcum* L.) reveals multiple mechanisms of genome evolution. *The Plant Journal*.

[146] Brooks SA, Huang L, Gill BS, Fellers JP (2002). Analysis of 106 kb of contiguous DNA sequence from the D genome of wheat reveals high gene density and a complex arrangement of genes related to disease resistance. *Genome*.

[147] Paux E, Roger D, Badaeva E (2006). Characterizing the composition and evolution of homoeologous genomes in hexaploid wheat through BAC-end sequencing on chromosome 3B. *The Plant Journal*.

[148] Sandhu D, Champoux JA, Bondareva SN, Gill KS (2001). Identification and physical localization of useful genes and markers to a major gene-rich region on wheat group 1S chromosomes. *Genetics*.

[149] Erayman M, Sandhu D, Sidhu D, Dilbirligi M, Baenziger PS, Gill KS (2004). Demarcating the gene-rich regions of the wheat genome. *Nucleic Acids Research*.

[150] Gill KS, Tsunewaki K (2005). Structural organization of the wheat genome. *Frontiers of Wheat Bioscience: The 100th Memorial Issue of Wheat Information Service*.

[151] Sidhu D, Gill KS (2005). Distribution of genes and recombination in wheat and other eukaryotes. *Plant Cell, Tissue and Organ Culture*.

[152] Barakat A, Carels N, Bernardi G (1997). The distribution of genes in the genomes of Gramineae. *Proceedings of the National Academy of Sciences of the United States of America*.

[153] Feuillet C, Keller B (1999). High gene density is conserved at syntenic loci of small and large grass genomes. *Proceedings of the National Academy of Sciences of the United States of America*.

[154] Sandhu D, Gill KS (2002). Gene-containing regions of wheat and the other grass genomes. *Plant Physiology*.

[155] Sandhu D, Gill KS (2002). Structural and functional organization of the ‘1S0.8 gene-rich region’ in the 
*Triticeae*. *Plant Molecular Biology*.

[156] Gupta PK, Kulwal PL, Rustgi S (2005). Wheat cytogenetics in the genomics era and its relevance to breeding. *Cytogenetic and Genome Research*.

[157] Wang ML, Leitch AR, Schwarzacher T, Heslop-Harrison JS, Moore G (1992). Construction of a chromosome-enriched *Hpall* library from flow-sorted wheat chromosomes. *Nucleic Acids Research*.

[158] Lee J-H, Arumuganathan K, Yen Y, Kaeppler S, Kaeppler H, Baenziger PS (1997). Root tip cell cycle synchronization and metaphase-chromosome isolation suitable for flow sorting in common wheat (*Triticum aestivum* L.). *Genome*.

[159] Vrána J, Kubalákova M, Simková H, Cíhalíková J, Lysák MA, Dolezel J (2000). Flow sorting of mitotic chromosomes in common wheat (*Triticum aestivum* L.). *Genetics*.

[160] Gill KS, Arumuganathan K, Lee J-H (1999). Isolating individual wheat (*Triticum aestivum*) chromosome arms by flow cytometric analysis of ditelosomic lines. *Theoretical and Applied Genetics*.

[161] Vega JM, Abbo S, Feldman M, Levy AA (1994). Chromosome painting in plants: in situ hybridization with a DNA probe from a specific microdissected chromosome arm of common wheat. *Proceedings of the National Academy of Sciences of the United States of America*.

[162] Chalhoub B, Belcram H, Caboche M (2004). Efficient cloning of plant genomes into bacterial artificial chromosome (BAC) libraries with larger and more uniform insert size. *Plant Biotechnology Journal*.

[163] Doležel J, Kubaláková M, Bartoš J, Macas J (2004). Flow cytogenetics and plant genome mapping. *Chromosome Research*.

[164] Kubaláková M, Vrána J, Číhalíková J, Šimková H, Doležel J (2002). Flow karyotyping and chromosome sorting in bread wheat (*Triticum aestivum* L.). *Theoretical and Applied Genetics*.

[165] Doležel J, Kubaláková M, Suchankova P, Tsunewaki K (2005). Flow cytogenetic analysis of the wheat genome. *Frontiers of Wheat Bioscience: The 100th Memorial Issue of Wheat Information Service*.

[166] Gill BS International genome research on wheat (IGROW).

[167] Gupta PK Ultrafast and low-cost DNA sequencing methods for applied genomics research.

[168] Wilson ID, Barker GLA, Beswick RW (2004). A transcriptomics resource for wheat functional genomics. *Plant Biotechnology Journal*.

[169] Wilson ID, Barker GL, Lu C (2005). Alteration of the embryo transcriptome of hexaploid winter wheat (*Triticum aestivum* cv. Mercia) during maturation and germination. *Functional and Integrative Genomics*.

[170] Poole R, Barker G, Wilson ID, Coghill JA, Edwards KJ (2007). Measuring global gene expression in polyploidy; a cautionary note from allohexaploid wheat. *Functional & Integrative Genomics*.

[171] McCartney CA, Somers DJ, Humphreys DG (2005). Mapping quantitative trait loci controlling agronomic traits in the spring wheat cross 
RL4452 × ‘AC Domain’. *Genome*.

[172] McCartney CA, Somers DJ, Lukow O (2006). QTL analysis of quality traits in the spring wheat cross RL4452 × ‘AC domain’. *Plant Breeding*.

[173] Fu D, Uauy C, Blechl A, Dubcovsky J (2007). RNA interference for wheat functional gene analysis. *Transgenic Research*.

[174] Salleh A (2006). Gene silencing yields high-fibre wheat.

[175] Mochida K, Yamazaki Y, Ogihara Y (2003). Discrimination of homoeologous gene expression in hexaploid wheat by SNP analysis of contigs grouped from a large number of expressed sequence tags. *Molecular Genetics and Genomics*.

[176] Schweizer P, Pokorny J, Schulze-Lefert P, Dudler R (2000). Double-stranded RNA interferes with gene function at the single-cell level in cereals. *The Plant Journal*.

[177] Christensen AB, Thordal-Christensen H, Zimmermann G (2004). The Germinlike protein GLP4 exhibits superoxide dismutase activity and is an important component of quantitative resistance in wheat and barley. *Molecular Plant-Microbe Interactions*.

[178] Loukoianov A, Yan L, Blechl A, Sanchez A, Dubcovsky J (2005). Regulation of *VRN-1* vernalization genes in normal and transgenic polyploid wheat. *Plant Physiology*.

[179] Regina A, Bird A, Topping D (2006). High-amylose wheat generated by RNA interference improves indices of large-bowel health in rats. *Proceedings of the National Academy of Sciences of the United States of America*.

[180] Travella S, Klimm TE, Keller B (2006). RNA interference-based gene silencing as an efficient tool for functional genomics in hexaploid bread wheat. *Plant Physiology*.

[181] Yao G, Zhang J, Yang L (2007). Genetic mapping of two powdery mildew resistance genes in einkorn (*Triticum monococcum* L.) accessions. *Theoretical and Applied Genetics*.

[182] Henikoff S, Till BJ, Comai L (2004). TILLING. Traditional mutagenesis meets functional genomics. *Plant Physiology*.

[183] Till BJ, Reynolds SH, Greene EA (2003). Large-scale discovery of induced point mutations with high-throughput TILLING. *Genome Research*.

[184] Slade AJ, Knauf VC (2005). TILLING moves beyond functional genomics into crop improvement. *Transgenic Research*.

[185] Slade AJ, Fuerstenberg SI, Loeffler D, Steine MN, Facciotti D (2005). A reverse genetic, nontransgenic approach to wheat crop improvement by TILLING. *Nature Biotechnology*.

[186] Devos KM, Gale MD (2000). Genome relationships: the grass model in current research. *The Plant Cell*.

[187] Wicker T, Yahiaoui N, Keller B (2007). Contrasting rates of evolution in *Pm3* loci from three wheat species and rice. *Genetics*.

[188] Huo N, Gu YQ, Lazo GR (2006). Construction and characterization of two BAC libraries from Brachypodium distachyon, a new model for grass genomics. *Genome*.

[189] Gale MD, Flintham JE, Devos KM (2002). Cereal comparative genetics and preharvest sprouting. *Euphytica*.

[190] Feuillet C, Penger A, Gellner K, Mast A, Keller B (2001). Molecular evolution of receptor-like kinase genes in hexaploid wheat. Independent evolution of orthologs after polyploidization and mechanisms of local rearrangements at paralogous loci. *Plant Physiology*.

[191] Chantret N, Cenci A, Sabot F, Anderson O, Dubcovsky J (2004). Sequencing of the *Triticum monococcum Hardness* locus reveals good microcolinearity with rice. *Molecular genetics and genomics*.

[192] Khlestkina EK, Pshenichnikova TA, Röder MS, Salina EA, Arbuzova VS, Börner A (2006). Comparative mapping of genes for glume colouration and pubescence in hexaploid wheat (*Triticum aestivum* L.). *Theoretical and Applied Genetics*.

[193] Devos KM, Atkinson MD, Chinoy CN, Liu CJ, Gale MD (1992). RFLP-based genetic map of the homoeologous group 3 chromosomes of wheat and rye. *Theoretical and Applied Genetics*.

[194] Stein N, Feuillet C, Wicker T, Schlagenhauf E, Keller B (2000). Subgenome chromosome walking in wheat: a 450-kb physical contig in *Triticum monococcum* L. spans the *Lr10* resistance locus in hexaploid wheat (*Triticum aestivum* L.). *Proceedings of the National Academy of Sciences of the United States of America*.

[195] Wicker T, Yahiaoui N, Guyot R (2003). Rapid genome divergence at orthologous low molecular weight glutenin loci of the A and A^m^ genomes of wheat. *The Plant Cell*.

[196] Isidore E, Scherrer B, Chalhoub B, Feuillet C, Keller B (2005). Ancient haplotypes resulting from extensive molecular rearrangements in the wheat A genome have been maintained in species of three different ploidy levels. *Genome Research*.

[197] Gu YQ, Salse J, Coleman-Derr D (2006). Types and rates of sequence evolution at the high-molecular-weight glutenin locus in hexaploid wheat and its ancestral genomes. *Genetics*.

[198] Guyot R, Yahiaoui N, Feuillet C, Keller B (2004). In silico comparative analysis reveals a mosaic conservation of genes within a novel colinear region in wheat chromosome 1AS and rice chromosome 5S. *Functional & Integrative Genomics*.

[199] Sorrells ME, La Rota M, Bermudez-Kandianis CE (2003). Comparative DNA sequence analysis of wheat and rice genomes. *Genome Research*.

[200] Singh NK, Raghuvanshi S, Srivastava SK (2004). Sequence analysis of the long arm of rice chromosome 11 for rice-wheat synteny. *Functional and Integrative Genomics*.

[201] Draper J, Mur LAJ, Jenkins G (2001). *Brachypodium distachyon*. A new model system for functional genomics in grasses. *Plant Physiology*.

[202] Hasterok R, Marasek A, Donnison IS (2006). Alignment of the genomes of *Brachypodium distachyon* and temperate cereals and grasses using bacterial artificial chromosome landing with fluorescence in *situ* hybridization. *Genetics*.

[203] Vogel JP, Gu YQ, Twigg P (2006). EST sequencing and phylogenetic analysis of the model grass *Brachypodium distachyon*. *Theoretical and Applied Genetics*.

[204] Bossolini E, Wicker T, Knobel PA, Keller B (2007). Comparison of orthologous loci from small grass genomes *Brachypodium* and rice: implications for wheat genomics and grass genome annotation. *The Plant Journal*.

[205] Xie Y, Ni Z, Yao Y, Yin Y, Zhang Q, Sun Q Analysis of differential cytosine methylation during seed development in wheat.

[206] Shitsukawa N, Tahira C, Kassai K-I (2007). Genetic and epigenetic alteration among three homoeologous genes of a class E MADS box gene in hexaploid wheat. *Plant Cell*.

[207] Nemoto Y, Kisaka M, Fuse T, Yano M, Ogihara Y (2003). Characterization and functional analysis of three wheat genes with homology to the *CONSTANS* flowering time gene in transgenic rice. *The Plant Journal*.

[208] Chantret N, Salse J, Sabot F (2005). Molecular basis of evolutionary events that shaped the hardness locus in diploid and polyploid wheat species (*Triticum and Aegilops*). *The Plant Cell*.

[209] Nomura T, Ishihara A, Yanagita RC, Endo TR, Iwamura H (2005). Three genomes differentially contribute to the biosynthesis of benzoxazinones in hexaploid wheat. *Proceedings of the National Academy of Sciences of the United States of America*.

[210] Comai L (2000). Genetic and epigenetic interactions in allopolyploid plants. *Plant Molecular Biology*.

[211] Chen ZJ, Ni Z (2006). Mechanisms of genomic rearrangements and gene expression changes in plant polyploids. *BioEssays*.

[212] Feldman M, Liu B, Segal G, Abbo S, Levy AA, Vega JM (1997). Rapid elimination of low-copy DNA sequences in polyploid wheat: a possible mechanism for differentiation of homoeologous chromosomes. *Genetics*.

[213] Liu B, Vega JM, Segal G, Abbo S, Rodova M, Feldman M (1998). Rapid genomic changes in newly synthesized amphiploids of *Triticum* and *Aegilops*—I: changes in low-copy noncoding DNA sequences. *Genome*.

[214] Liu B, Vega JM, Feldman M (1998). Rapid genomic changes in newly synthesized amphiploids of *Triticum* and *Aegilops*—II: changes in low-copy coding DNA sequences. *Genome*.

[215] Xiong LZ, Xu CG, Maroof MAS, Zhang Q (1999). Patterns of cytosine methylation in an elite rice hybrid and its parental lines, detected by a methylation-sensitive amplification polymorphism technique. *Molecular and General Genetics*.

[216] Shaked H, Kashkush K, Özkan H, Feldman M, Levy AA (2001). Sequence elimination and cytosine methylation are rapid and reproducible responses of the genome to wide hybridization and allopolyploidy in wheat. *Plant Cell*.

[217] Kashkush K, Feldman M, Levy AA (2002). Gene loss, silencing and activation in a newly synthesized wheat allotetraploid. *Genetics*.

[218] Levy AA, Feldman M (2004). Genetic and epigenetic reprogramming of the wheat genome upon allopolyploidization. *Biological Journal of the Linnean Society*.

[219] He P, Friebe BR, Gill BS, Zhou J-M (2003). Allopolyploidy alters gene expression in the highly stable hexaploid wheat. *Plant Molecular Biology*.

[220] Kulwal PL, Singh R, Balyan HS, Gupta PK (2004). Genetic basis of pre-harvest sprouting tolerance using single-locus and two-locus QTL analyses in bread wheat. *Functional & Integrative Genomics*.

[221] Kulwal PL, Kumar N, Kumar A, Gupta RK, Balyan HS, Gupta PK (2005). Gene networks in hexaploid wheat: interacting quantitative trait loci for grain protein content. *Functional & Integrative Genomics*.

[222] Kumar N, Kulwal PL, Balyan HS, Gupta PK (2007). QTL mapping for yield and yield contributing traits in two mapping populations of bread wheat. *Molecular Breeding*.

[223] Langridge P, Lagudah ES, Holton TA, Appels R, Sharp PJ, Chalmers KJ (2001). Trends in genetic and genome analyses in wheat: a review. *Australian Journal of Agricultural Research*.

[224] Jahoor A, Eriksen L, Backes G, Gupta PK, Varshney RK (2004). QTLs and genes for disease resistance in barley and wheat. *Cereal Genomics*.

[225] Tuberosa R, Salvi S, Gupta PK, Varshney RK (2004). QTLs and genes for tolerance to abiotic stresses in cereals. *Cereal Genomics*.

[226] Gupta PK, Rustgi S, Kumar N (2006). Genetic and molecular basis of grain size and grain number and its relevance to grain productivity in higher plants. *Genome*.

[227] Li W, Gill BS, Gupta PK, Varshney RK (2004). Genomics for cereal improvement. *Cereal Genomics*.

[228] Tanksley SD, Nelson JC (1996). Advanced backcross QTL analysis: a method for the simultaneous discovery and transfer of valuable QTLs from unadapted germplasm into elite breeding lines. *Theoretical and Applied Genetics*.

[229] Huang XQ, Kempf H, Canal MW, Röder MS (2004). Advanced backcross QTL analysis in progenies derived from a cross between a German elite winter wheat variety and a synthetic wheat (*Triticum aestivum* L.). *Theoretical and Applied Genetics*.

[230] Kunert A, Naz AA, Dedeck O, Pillen K, Léon J (2007). AB-QTL analysis in winter wheat—I: synthetic hexaploid wheat (*T. turgidum* ssp. *dicoccoides* × *T. tauschii*) as a source of favourable alleles for milling and baking quality traits. *Theoretical and Applied Genetics*.

[231] Amiour N, Merlino M, Leroy P, Branlard G (2003). Chromosome mapping and identification of amphiphilic proteins of hexaploid wheat kernels. *Theoretical and Applied Genetics*.

[232] Flavell RB, Bennett MD, Seal AG, Hutchinson J, Lupton FGH (1987). Chromosome structure and organisation. *Wheat Breeding, Its Scientific Basis*.

[233] Kimber G, Swaminathan MS, Gupta PK, Sinha U (1983). The B genome of wheat: the present status. *Cytogenetics of Crop Plants*.

[234] Kimber G, Sears ER, Heyne EG (1987). Evolution in the genus *Triticum* and the origin of cultivated wheat. *Wheat and Wheat Improvement*.

[235] Feldman M, Lupton FGH, Miller TE, Smartt J, Simmonds NW (1995). Wheats. *Evolution of Crops*.

[236] Gill BS, Friebe B, Curtis BC, Rajaram S, Macpherson HG (2002). Cytogenetics, phylogeny and evolution of cultivated wheats. *Bread Wheat, Improvement and Production*.

[237] Yen Y, Baenziger PS, Morris R, Jauhar PP (1996). Genomic constitution of bread wheat: current status. *Methods of Genome Analysis in Plants*.

[238] Levy AA, Feldman M (2002). The impact of polyploidy on grass genome evolution. *Plant Physiology*.

[239] Caldwell KS, Dvorak J, Lagudah ES (2004). Sequence polymorphism in polyploid wheat and their D-genome diploid ancestor. *Genetics*.

[240] Huang S, Sirikhachornkit A, Su X (2002). Genes encoding plastid acetyl-CoA carboxylase and 3-phosphoglycerate kinase of the *Triticum/Aegilops* complex and the evolutionary history of polyploid wheat. *Proceedings of the National Academy of Sciences of the United States of America*.

[241] Maestra B, Naranjo T (1998). Homoeologous relationships of *Aegilops speltoides* chromosomes to bread wheat. *Theoretical and Applied Genetics*.

[242] Nevo E, Korol AB, Beiles A, Fahima T (2002). *Evolution of Wild Emmer and Wheat Improvement*.

[243] Blake NK, Lehfeldt BR, Lavin M, Talbert LE (1999). Phylogenetic reconstruction based on low copy DNA sequence data in an allopolyploid: the B genome of wheat. *Genome*.

[244] Kilian B, Özkan H, Deusch O (2007). Independent wheat B and G genome origins in outcrossing *Aegilops* progenitor haplotypes. *Molecular Biology and Evolution*.

[245] Talbert LE, Blake NK Comparative DNA sequence analysis and the origin of wheat.

[246] Sabot F, Laubin B, Amilhat L, Leroy P, Sourdille P, Bernard M Evolution history of the *Triticum* sp. through the study of transposable elements.

[247] Schulman AH, Gupta PK, Varshney RK, Gupta PK, Varshney RK (2004). Organization of retrotransposons and microsatellites in cereal genomes. *Cereal Genomics*.

[248] Schachermayr G, Siedler H, Gale MD, Winzeler H, Winzeler M, Keller B (1994). Identification and localization of molecular markers linked to the *Lr9* leaf rust resistance 
gene of wheat. *Theoretical and Applied Genetics*.

[249] Feuillet C, Messmer M, Schachermayr G, Keller B (1995). Genetic and physical characterization of the *LR1* leaf rust resistance locus in wheat (*Triticum aestivum* L.). *Molecular and General Genetics*.

[250] Schachermayr GM, Messmer MM, Feuillet C, Winzeler H, Winzeler M, Keller B (1995). Identification of molecular markers linked to the *Agropyron elongatum*-derived leaf rust resistance gene *Lr24* in wheat. *Theoretical and Applied Genetics*.

[251] Schachermayr G, Feuillet C, Keller B (1997). Molecular markers for the detection of the wheat leaf rust resistance gene *Lr10* in diverse genetic backgrounds. *Molecular Breeding*.

[252] Naik S, Gill KS, Prakasa Rao VS (1998). Identification of a STS marker linked to the *Aegilops speltoides*-derived leaf rust resistance gene *Lr28* in wheat. *Theoretical and Applied Genetics*.

[253] Sacco F, Suárez EY, Naranjo T (1998). Mapping of the leaf rust resistance gene *Lr3* on chromosome 6B of Sinvalocho MA wheat. *Genome*.

[254] Seyfarth R, Feuillet C, Schachermayr G, Winzeler M, Keller B (1999). Development of a molecular marker for the adult plant leaf rust resistance gene *Lr35* in wheat. *Theoretical and Applied Genetics*.

[255] Helguera M, Khan IA, Dubcovsky J (2000). Development of PCR markers for the wheat leaf rust resistance gene *L*
*r*47. *Theoretical and Applied Genetics*.

[256] Aghaee-Sarbarzeh M, Singh H, Dhaliwal HS (2001). A microsatellite marker linked to leaf rust resistance transferred from *Aegilops triuncialis* into hexaploid wheat. *Plant Breeding*.

[257] Prins R, Groenewald JZ, Marais GF, Snape JW, Koebner RMD (2001). AFLP and STS tagging of *Lr19*, a gene conferring resistance to leaf rust in wheat. *Theoretical and Applied Genetics*.

[258] Raupp WJ, Singh S, Brown-Guedira GL, Gill BS (2001). Cytogenetic and molecular mapping of the leaf rust resistance gene *Lr39* in wheat. *Theoretical and Applied Genetics*.

[259] Seah S, Bariana H, Jahier J, Sivasithamparam K, Lagudah ES (2001). The introgressed segment carrying rust resistance genes *Yr17*, *Lr37* and *Sr38* in wheat can be assayed by a cloned disease resistance gene-like sequence. *Theoretical and Applied Genetics*.

[260] Neu C, Stein N, Keller B (2002). Genetic mapping of the *Lr20-Pm1* resistance locus reveals suppressed recombination on chromosome arm 7AL in hexaploid wheat. *Genome*.

[261] Cherukuri DP, Gupta SK, Charpe A (2003). Identification of a molecular marker linked to an *Agropyron elongatum*-derived gene *Lr19* for leaf rust resistance in wheat. *Plant Breeding*.

[262] Ling H-Q, Qiu J, Singh RP, Keller B (2004). Identification and genetic characterization of an *Aegilops tauschii* ortholog of the wheat leaf rust disease resistance gene *L*
*r*1. *Theoretical and Applied Genetics*.

[263] Cherukuri DP, Gupta SK, Charpe A (2005). Molecular mapping of *Aegilops speltoides* derived leaf rust resistance gene *Lr28* in wheat. *Euphytica*.

[264] Spielmeyer W, McIntosh RA, Kolmer J, Lagudah ES (2005). Powdery mildew resistance and *Lr34/Yr18* genes for durable resistance to leaf and stripe rust cosegregate at a locus on the short arm of chromosome 7D of wheat. *Theoretical and Applied Genetics*.

[265] Hiebert CW, Thomas JB, McCallum BD (2005). Locating the broad-spectrum wheat leaf rust resistance gene *L*
*r*52(*L*
*r*
*W*) to chromosome 5B by a new cytogenetic method. *Theoretical and Applied Genetics*.

[266] Gupta SK, Charpe A, Prabhu KV, Haque QMR (2006). Identification and validation of molecular markers linked to the leaf rust resistance gene *L*
*r*19 in wheat. *Theoretical and Applied Genetics*.

[267] Bossolini E, Krattinger SG, Keller B (2006). Development of simple sequence repeat markers specific for the *Lr34* resistance region of wheat using sequence information from rice and *Aegilops tauschii*. *Theoretical and Applied Genetics*.

[268] Hiebert CW, Thomas JB, Somers DJ, McCallum BD, Fox SL (2007). Microsatellite mapping of adult-plant leaf rust resistance gene *L*
*r*22*a* in wheat. *Theoretical and Applied Genetics*.

[269] Qiu J-W, Schürch AC, Yahiaoui N (2007). Physical mapping and identification of a candidate for 
the leaf rust resistance gene *Lr1* of wheat. *Theoretical and Applied Genetics*.

[270] Obert DE, Fritz AK, Moran JL, Singh S, Rudd JC, Menz MA (2005). Identification and molecular tagging of a gene from PI 289824 conferring resistance to leaf rust (*Puccinia triticina*) in wheat. *Theoretical and Applied Genetics*.

[271] Schnurbusch T, Paillard S, Schori A (2004). Dissection of quantitative and durable leaf rust resistance in Swiss winter wheat reveals a major resistance QTL in the *Lr34* chromosomal region. *Theoretical and Applied Genetics*.

[272] Leonova IN, Laikova LI, Popova OM, Unger O, Börner A, Röder MS (2007). Detection of quantitative trait loci for leaf rust resistance in wheat—*T. timopheevii/T. tauschii* introgression lines. *Euphytica*.

[273] Singh RP, Nelson JC, Sorrells ME (2000). Mapping *Yr28* and other genes for resistance to stripe rust in wheat. *Crop Science*.

[274] Sun GL, Fahima T, Korol AB (1997). Identification of molecular markers linked to the *Yr15* stripe rust resistance gene of wheat originated in wild emmer wheat, *Triticum dicoccoides*. *Theoretical and Applied Genetics*.

[275] Peng JH, Fahima T, Röder MS (1999). Microsatellite tagging of the stripe-rust resistance gene *YrH52* derived from wild emmer wheat, *Triticum dicoccoides*, and suggestive negative crossover interference on chromosome 1B. *Theoretical and Applied Genetics*.

[276] Börner A, Röder MS, Unger O, Meinel A (2000). The detection and molecular mapping of a major gene for non-specific adult-plant disease resistance against stripe rust (*Puccinia striiformis*) in wheat. *Theoretical and Applied Genetics*.

[277] Peng JH, Fahima T, Röder MS (2001). High-density molecular map of chromosome region harboring stripe-rust resistance genes 
*YrH52* and *Yr15* derived from wild emmer wheat, *Triticum dicoccoides*. *Genetica*.

[278] Shi ZX, Chen XM, Line RF, Leung H, Wellings CR (2001). Development of resistance gene analog polymorphism markers for the *Yr9* gene resistance to wheat stripe rust. *Genome*.

[279] Ma J, Zhou R, Dong Y, Wang L, Wang X, Jia J (2001). Molecular mapping and detection of the yellow rust resistance gene *Yr26* in wheat transferred from *Triticum turgidum* L. using microsatellite markers. *Euphytica*.

[280] Wang L, Ma J, Zhou R, Wang X, Jia J (2002). Molecular tagging of the yellow rust resistance gene *Yr10* in common wheat, P.I.178383 (*Triticum aestivum* L.). *Euphytica*.

[281] Uauy C, Brevis JC, Chen X (2005). High-temperature adult-plant (HTAP) stripe rust resistance gene *Yr36* from *Triticum turgidum* ssp. *dicoccoides* is closely linked to the grain protein content locus Gpc-B1. *Theoretical and Applied Genetics*.

[282] Li GQ, Li ZF, Yang WY (2006). Molecular mapping of stripe rust resistance gene *Y*
*r*
*C*
*H*42 in Chinese wheat cultivar Chuanmai 42 and its allelism with *Y*
*r*24 and *Y*
*r*26. *Theoretical and applied genetics*.

[283] Li ZF, Zheng TC, He ZH (2006). Molecular tagging of stripe rust resistance gene *Y*
*r*
*Z*
*H*84 in Chinese wheat line Zhou 8425B. *Theoretical and Applied Genetics*.

[284] Bariana HS, Parry N, Barclay IR (2006). Identification and characterization of stripe rust resistance gene *Yr34* in common wheat. *Theoretical and Applied Genetics*.

[285] Wang C, Zhang Y, Han D (2008). SSR and STS markers for wheat stripe rust resistance gene *Yr26*. *Euphytica*.

[286] Mallard S, Gaudet D, Aldeia A (2005). Genetic analysis of durable resistance to yellow rust in bread wheat. *Theoretical and Applied Genetics*.

[287] Christiansen MJ, Feenstra B, Skovgaard IM, Andersen SB (2006). Genetic analysis of resistance to yellow rust in hexaploid wheat using a mixture model for multiple crosses. *Theoretical and Applied Genetics*.

[288] Paull JG, Pallotta MA, Langridge P, The TT (1994). RFLP markers associated with *Sr22* and recombination between chromosome 7A of bread wheat and the diploid species Triticum boeoticum. *Theoretical and Applied Genetics*.

[289] Spielmeyer W, Sharp PJ, Lagudah ES (2003). Identification and validation of markers linked to broad-spectrum stem rust resistance gene *Sr2* in wheat (*Triticum aestivum* L.). *Crop Science*.

[290] Cuthbert PA, Somers DJ, Brulé-Babel A (2007). Mapping of *Fhb2* on chromosome 6BS: a gene controlling Fusarium head blight field resistance in bread wheat (*Triticum aestivum* L.). *Theoretical and Applied Genetics*.

[291] Bourdoncle W, Ohm HW (2003). Quantitative trait loci for resistance to Fusarium head blight in recombinant inbred wheat lines from the cross huapei 57-2/Patterson. *Euphytica*.

[292] del Blanco IA, Frohberg RC, Stack RW, Berzonsky WA, Kianian SF (2003). Detection of QTL linked to Fusarium head blight resistance in Sumai 3-derived North Dakota bread wheat lines. *Theoretical and Applied Genetics*.

[293] Lin F, Kong ZX, Zhu HL (2004). Mapping QTL associated with resistance to Fusarium head blight in the Nanda2419 × Wangshuibai population—I: type II resistance. *Theoretical and Applied Genetics*.

[294] Lin F, Xue SL, Zhang ZZ (2006). Mapping QTL associated with resistance to Fusarium head blight in the Nanda2419 × Wangshuibai population—II: type I resistance. *Theoretical and Applied Genetics*.

[295] Paillard S, Schnurbusch T, Tiwari R (2004). QTL analysis of resistance to Fusarium head blight in Swiss winter 
wheat (*Triticum aestivum* L.). *Theoretical and Applied Genetics*.

[296] Gilsinger J, Kong L, Shen X, Ohm H (2005). DNA markers associated with low Fusarium head blight incidence and narrow flower opening in wheat. *Theoretical and Applied Genetics*.

[297] Jia G, Chen P, Qin G (2005). QTLs for Fusarium head blight response in a wheat DH population of Wangshuibai/Alondra‘s’. *Euphytica*.

[298] Chen X, Faris JD, Hu J (2007). Saturation and comparative mapping of a major Fusarium head blight resistance QTL in tetraploid wheat. *Molecular Breeding*.

[299] Jiang G-L, Dong Y, Shi J, Ward RW (2007). QTL analysis of resistance to Fusarium head blight in the novel wheat germplasm CJ 9306—II: resistance to deoxynivalenol accumulation and grain yield loss. *Theoretical and Applied Genetics*.

[300] Klahr A, Zimmermann G, Wenzel G, Mohammedler V (2007). Effects of environment, disease progress, plant height and heading date on the detection of QTLs for resistance to Fusarium head blight in an European winter wheat cross. *Euphytica*.

[301] Shen X, Ohm H (2007). Molecular mapping of *Thinopyrum*-derived *Fusarium* head blight resistance in common wheat. *Molecular Breeding*.

[302] Zhou W, Kolb FL, Bai G, Shaner G, Domier LL (2002). Genetic analysis of scab resistance QTL in wheat with microsatellite and AFLP markers. *Genome*.

[303] Zhou W-C, Kolb FL, Bai G-H, Domier LL, Boze LK, Smith NJ (2003). Validation of a major QTL for scab resistance with SSR markers and use of marker-assisted selection in wheat. *Plant Breeding*.

[304] Hartl L, Weiss H, Stephan U, Zeller FJ, Jahoor A (1995). Molecular identification of powdery mildew resistance genes in common wheat (*Triticum aestivum* L.). *Theoretical and Applied Genetics*.

[305] Jia J, Devos KM, Chao S, Miller TE, Reader SM, Gale MD (1996). RFLP-based maps of the homoeologous group-6 chromosomes of wheat and their application in the tagging of *P*
*m*12, a powdery mildew resistance gene transferred from *Aegilops speltoides* to wheat. *Theoretical and Applied Genetics*.

[306] Liu Z, Sun Q, Ni Z, Yang T (1999). Development of SCAR markers linked to the *P*
*m*21 gene conferring resistance to powdery mildew in common wheat. *Plant Breeding*.

[307] Sourdille P, Robe P, Tixier M-H, Doussinault G, Pavoinc M-T, Bernard M (1999). Location of *Pm3g*, a powdery mildew resistance allele in wheat, by using a monosomic analysis and by identifying associated molecular markers. *Euphytica*.

[308] Huang XQ, Hsam SLK, Zeller FJ, Wenzel G, Mohammedler V (2000). Molecular mapping of the wheat powdery mildew resistance gene *P*
*m*24 and marker validation for molecular breeding. *Theoretical and Applied Genetics*.

[309] Rong JK, Millet E, Manisterski J, Feldman M (2000). A new powdery mildew resistance gene: introgression from wild emmer into common wheat and RFLP-based mapping. *Euphytica*.

[310] Tao WJ, Liu D, Liu JY, Feng Y, Chen P (2000). Genetic mapping of the powdery mildew resistance gene *Pm6* in wheat by RFLP analysis. *Theoretical and Applied Genetics*.

[311] Järve K, Peusha HO, Tsymbalova J, Tamm S, Devos KM, Enno TM (2000). Chromosomal location of a *Triticum timopheevii*-derived powdery mildew resistance gene transferred to common wheat. *Genome*.

[312] Mohammedler V, Hsam SLK, Zeller FJ, Wenzel G (2001). An STS marker distinguishing the rye-derived powdery mildew resistance alleles at the *Pm8/Pm17* locus of common wheat. *Plant Breeding*.

[313] Bougot Y, Lemoine J, Pavoine MT, Barloy D, Doussinault G (2002). Identification of a microsatellite marker associated with *Pm3* resistance alleles to powdery mildew in wheat. *Plant Breeding*.

[314] Zeller FJ, Kong L, Hartl L, Mohammedler V, Hsam SLK (2002). Chromosomal location of genes for resistance to powdery mildew in common wheat (*Triticum aestivum* L. em Thell.) 7. Gene *Pm29* in line Pova. *Euphytica*.

[315] Liu Z, Sun Q, Ni Z, Nevo E, Yang T (2002). Molecular characterization of a novel powdery mildew resistance gene *P*
*m*30 in wheat originating from wild emmer. *Euphytica*.

[316] Alberto C, Renato D, Antonio TO, Carla C, Marina P, Enrico P (2003). Genetic analysis of the *Aegilops longissima* 3S chromosome carrying the *Pm13* resistance gene. *Euphytica*.

[317] Ma Z-Q, Wei J-B, Cheng S-H (2004). PCR-based markers for the powdery mildew resistance gene *Pm4a* in wheat. *Theoretical and Applied Genetics*.

[318] Qiu YC, Zhou RH, Kong XY, Zhang SS, Jia JZ (2005). Microsatellite mapping of a *Triticum urartu* Tum. derived powdery mildew resistance gene transferred to common wheat (*Triticum aestivum* L.). *Theoretical and Applied Genetics*.

[319] Miranda LM, Murphy JP, Marshall D, Leath S (2006). *Pm34*: a new powdery mildew resistance gene transferred from 
*Aegilops tauschii* Coss. to common wheat (*Triticum aestivum* L.). *Theoretical and Applied Genetics*.

[320] Zhu Z, Zhou R, Kong X, Dong Y, Jia J (2006). Microsatellite marker identification of a *Triticum aestivum*—*Aegilops umbellulata* substitution line with powdery mildew resistance. *Euphytica*.

[321] Miranda LM, Murphy JP, Marshall D, Cowger C, Leath S (2007). Chromosomal location of *Pm35*, a novel *Aegilops tauschii* derived powdery mildew resistance gene introgressed into common wheat (*Triticum aestivum* L.). *Theoretical and Applied Genetics*.

[322] Nematollahi G, Mohammedler V, Wenzel G, Zeller FJ, Hsam SLK (2008). Microsatellite mapping of powdery mildew resistance allele *Pm5d* 
from common wheat line IGV1-455. *Euphytica*.

[323] Song W, Xie H, Liu Q (2007). Molecular identification of *Pm12*-carrying introgression lines in wheat using genomic and EST-SSR markers. *Euphytica*.

[324] Chantret N, Sourdille P, Röder M, Tavaud M, Bernard M, Doussinault G (2000). Location and mapping of the powdery mildew resistance gene *MlRE* and detection of a resistance QTL by bulked segregant analysis (BSA) with microsatellites in wheat. *Theoretical and Applied Genetics*.

[325] Xie C, Sun Q, Ni Z, Yang T, Nevo E, Fahima T (2003). Chromosomal location of a *Triticum dicoccoides*-derived powdery mildew resistance gene in common wheat by using microsatellite markers. *Theoretical and Applied Genetics*.

[326] Singrün Ch, Hsam SLK, Zeller FJ, Wenzel G, Mohammedler V (2004). Localization of a novel recessive powdery mildew resistance gene from common wheat line RD30 in the terminal region of chromosome 7AL. *Theoretical and Applied Genetics*.

[327] Keller M, Keller B, Schachermayr G (1999). Quantitative trait loci for resistance against powdery mildew in a segregating wheat × spelt population. *Theoretical and Applied Genetics*.

[328] Liu S, Griffey CA, Saghai Maroof MA (2001). Identification of molecular markers associated with adult plant resistance to powdery mildew in common wheat cultivar Massey. *Crop Science*.

[329] Mingeot D, Chantret N, Baret PV (2002). Mapping QTL involved in adult plant resistance to powdery mildew in the winter wheat line RE714 in two susceptible genetic backgrounds. *Plant Breeding*.

[330] Bougot Y, Lemoine J, Pavoine MT (2006). A major QTL effect controlling resistance to powdery mildew in winter wheat at the adult plant stage. *Plant Breeding*.

[331] Tucker DM, Griffey CA, Liu S, Brown-Guedira G, Marshall DS, Maroof MAS (2007). Confirmation of three quantitative trait loci conferring adult plant resistance to powdery mildew in two winter wheat populations. *Euphytica*.

[332] Laroche A, Demeke T, Gaudet DA, Puchalski B, Frick M, McKenzie R (2000). Development of a PCR marker for rapid identification of the *B*
*t*-10 gene for common bunt resistance in wheat. *Genome*.

[333] Fofana B, Humphreys DG, Cloutier S, McCartney CA, Somers DJ (2008). Mapping quantitative trait loci controlling common bunt resistance in a doubled haploid population derived from the spring wheat cross RL4452 × AC Domain. *Molecular Breeding*.

[334] Faris JD, Anderson JA, Francl LJ, Jordahl JG (1997). RFLP mapping of resistance to chlorosis induction by *Pyrenophora tritici-repentis* in wheat. *Theoretical and Applied Genetics*.

[335] Singh PK, Mergoum M, Adhikari TB, Kianian SF, Elias EM (2007). Chromosomal location of genes for seedling resistance to tan spot and *Stagonospora nodorum* blotch in tetraploid wheat. *Euphytica*.

[336] Tadesse W, Schmolke M, Hsam SLK, Mohammedler V, Wenzel G, Zeller FJ (2007). Molecular mapping of resistance genes to tan spot [*Pyrenophora tritici-repentis* race 1] in synthetic wheat lines. *Theoretical and Applied Genetics*.

[337] Arraiano LS, Worland AJ, Ellerbrook C, Brown JKM (2001). Chromosomal location of a gene for resistance to septoria tritici blotch (*Mycosphaerella graminicola*) in the hexaploid wheat ‘Synthetic 6x’. *Theoretical and Applied Genetics*.

[338] Simón MR, Ayala FM, Cordo CA, Röder MS, Börner A (2004). Molecular mapping of quantitative trait loci determining resistance to septoria tritici blotch caused by *Mycosphaerella graminicola* in wheat. *Euphytica*.

[339] Ayala L, Henry M, van Ginkel M, Singh R, Keller B, Khairallah M (2002). Identification of QTLs for BYDV tolerance in bread wheat. *Euphytica*.

[340] Aguilar V, Stamp P, Winzeler M (2005). Inheritance of field resistance to *Stagonospora nodorum* leaf and glume blotch and correlations with other morphological traits in hexaploid wheat (*Triticum aestivum* L.). *Theoretical and Applied Genetics*.

[341] Talbert LE, Bruckner PL, Smith LY, Sears R, Martin TJ (1996). Development of PCR markers linked to resistance to wheat streak mosaic virus in wheat. *Theoretical and Applied Genetics*.

[342] Khan AA, Bergstrom GC, Nelson JC, Sorrells ME (2000). Identification of RFLP markers for resistance to wheat spindle streak mosaic bymovirus (WSSMV) disease. *Genome*.

[343] Liu W, Nie H, Wang S (2005). Mapping a resistance gene in wheat cultivar Yangfu 9311 to 
yellow mosaic virus, using microsatellite markers. *Theoretical and Applied Genetics*.

[344] de la Peña RC, Murray TD, Jones SS (1997). Identification of an RFLP interval containing *Pch2* on chromosome 7AL in wheat. *Genome*.

[345] Huguet-Robert V, Dedryver F, Röder MS (2001). Isolation of a chromosomally engineered durum wheat line carrying the *Aegilops ventricosa*
*P*
*c*
*h*1 gene for resistance to eyespot. *Genome*.

[346] Groenewald JZ, Marais AS, Marais GF (2003). Amplified fragment length polymorphism-derived microsatellite sequence linked to the *P*
*c*
*h*1 and *E*
*p*-*D*1 loci in common wheat. *Plant Breeding*.

[347] Weng Y, Lazar MD (2002). Amplified fragment length polymorphism- and simple sequence repeat-based molecular tagging and mapping of greenbug resistance gene *Gb3* in wheat. *Plant Breeding*.

[348] Boyko E, Starkey S, Smith M (2004). Molecular genetic mapping of *Gby*, a new greenbug resistance gene in bread wheat. *Theoretical and Applied Genetics*.

[349] Weng Y, Li W, Devkota RN, Rudd JC (2005). Microsatellite markers associated with two *Aegilops tauschii*-derived greenbug resistance loci in wheat. *Theoretical and Applied Genetics*.

[350] Zhu LC, Smith CM, Fritz A, Boyko E, Voothuluru P, Gill BS (2005). Inheritance and molecular mapping of new greenbug resistance genes in wheat germplasms derived from *Aegilops tauschii*. *Theoretical and Applied Genetics*.

[351] Ma Z-Q, Gill BS, Sorrells ME, Tanksley SD (1993). RELP markers linked to two Hessian fly-resistance genes in wheat (*Triticum aestivum* L.) from *Triticum tauschii* (coss.) Schmal. *Theoretical and Applied Genetics*.

[352] Dweikat I, Ohm HW, Mackenzie S, Patterson F, Cambron S, Ratcliffe R (1994). Association of a DNA marker with Hessian fly resistance gene *H9* in wheat. *Theoretical and Applied Genetics*.

[353] Dweikat I, Ohm HW, Patterson F, Cambron S (1997). Identification of RAPD markers for 11 Hessian fly resistance genes in wheat. *Theoretical and Applied Genetics*.

[354] Seo YW, Johnson JW, Jarret RL (1997). A molecular marker associated with the *H21* Hessian fly resistance gene in wheat. *Molecular Breeding*.

[355] Dweikat I, Zhang W, Ohm HW (2002). Development of STS markers linked to Hessian fly resistance gene *H6* in wheat. *Theoretical and Applied Genetics*.

[356] Liu XM, Gill BS, Chen M-S (2005). Hessian fly resistance gene *H*13 is mapped to a distal cluster of resistance genes in chromosome 6DS of wheat. *Theoretical and Applied Genetics*.

[357] Wang T, Xu SS, Harris MO, Hu J, Liu L, Cai X (2006). Genetic characterization and molecular mapping of Hessian fly resistance genes derived from *Aegilops tauschii* in synthetic wheat. *Theoretical and Applied Genetics*.

[358] Zhao HX, Liu XM, Chen M-S (2006). *H22*, a major resistance gene to the Hessian fly (*Mayetiola destructor*), is mapped to the distal region of wheat chromosome 1DS. *Theoretical and Applied Genetics*.

[359] Kong L, Cambron SE, Ohm HW (2008). Hessian fly resistance genes *H*16 and *H*17 are mapped to a resistance gene cluster in the distal region of chromosome 1AS in wheat. *Molecular Breeding*.

[360] Liu XM, Smith CM, Gill BS, Tolmay V (2001). Microsatellite markers linked to six Russian wheat aphid resistance genes in wheat. *Theoretical and Applied Genetics*.

[361] Miller CA, Altinkut A, Lapitan NLV (2001). A microsatellite marker for tagging *Dn2*, a wheat gene 
conferring resistance to the Russian wheat aphid. *Crop Science*.

[362] Liu XM, Smith CM, Gill BS (2002). Identification of microsatellite markers linked to Russian wheat aphid resistance genes *Dn4* and *Dn6*. *Theoretical and Applied Genetics*.

[363] Williams KJ, Fisher JM, Langridge P (1994). Identification of RFLP markers linked to the cereal cyst nematode resistance gene (*Cre*) in wheat. *Theoretical and Applied Genetics*.

[364] Jahier J, Abelard P, Tanguy AM (2001). The *Aegilops ventricosa* segment on chromosome 2AS of the wheat cultivar ‘VPM1’ carries the cereal cyst nematode resistance gene *C*
*r*
*e*5. *Plant Breeding*.

[365] Ogbonnaya FC, Seah S, Delibes A (2001). Molecular-genetic characterisation of a new nematode resistance gene in wheat. *Theoretical and Applied Genetics*.

[366] Barloy D, Lemoine J, Dredryver F, Jahier J (2000). Molecular markers linked to the *Aegilops variabilis*-derived root-knot nematode resistance gene *Rkn-mn1* in wheat. *Plant Breeding*.

[367] Williams KJ, Taylor SP, Bogacki P, Pallotta M, Bariana HS, Wallwork H (2002). Mapping of the root lesion nematode (*Pratylenchus neglectus*) resistance gene *Rlnn1* in wheat. *Theoretical and Applied Genetics*.

[368] Kato K, Nakamura W, Tabiki T, Miura H, Sawada S (2001). Detection of loci controlling seed dormancy on group 4 chromosomes of wheat and comparative mapping with rice and barley genomes. *Theoretical and Applied Genetics*.

[369] Flintham J, Adlam R, Bassoi M, Holdsworth M, Gale MD (2002). Mapping genes for resistance to sprouting damage in wheat. *Euphytica*.

[370] Miura H, Sato N, Kato K, Amano Y (2002). Detection of chromosomes carrying genes for seed dormancy of wheat using the backcross reciprocal monosomic method. *Plant Breeding*.

[371] Osa M, Kato K, Mori M, Shindo C, Torada A, Miura H (2003). Mapping QTLs for seed dormancy and the *Vp1* homologue on chromosome 3A in wheat. *Theoretical and Applied Genetics*.

[372] Kulwal PL, Kumar N, Gaur A (2005). Mapping of a major QTL for pre-harvest sprouting tolerance on chromosome 3A in bread wheat. *Theoretical and Applied Genetics*.

[373] Mares D, Mrva K, Cheong J (2005). A QTL located on chromosome 4A associated with dormancy in 
white- and red-grained wheats of diverse origin. *Theoretical and Applied Genetics*.

[374] Joppa LR, Du C, Hart GE, Hareland GA (1997). Mapping gene(s) for grain protein in tetraploid wheat (*Triticum turgidum* L.) using a population of recombinant inbred chromosome lines. *Crop Science*.

[375] Prasad M, Kumar N, Kulwal PL (2003). QTL analysis for grain protein content using SSR markers 
and validation studies using NILs in bread wheat. *Theoretical and Applied Genetics*.

[376] Blanco A, Simeone R, Gadaleta A (2006). Detection of QTLs for grain protein content in durum wheat. *Theoretical and Applied Genetics*.

[377] Parker GD, Chalmers KJ, Rathjen AJ, Langridge P (1998). Mapping loci associated with flour colour in wheat (*Triticum aestivum* L.). *Theoretical and Applied Genetics*.

[378] Parker GD, Chalmers KJ, Rathjen AJ, Langridge P (1999). Mapping loci associated with milling yield in wheat (*Triticum aestivum* L.). *Molecular Breeding*.

[379] Perretant MR, Cadalen T, Charmet G (2000). QTL analysis of bread-making quality in wheat using a doubled haploid population. *Theoretical and Applied Genetics*.

[380] Charmet G, Robert N, Branlard G, Linossier L, Martre P, Triboï E (2005). Genetic analysis of dry matter and nitrogen accumulation and protein composition in wheat kernels. *Theoretical and Applied Genetics*.

[381] Ma W, Appels R, Bekes F, Larroque O, Morell MK, Gale KR (2005). Genetic characterisation of dough rheological properties in a wheat doubled haploid population: additive genetic effects and epistatic interactions. *Theoretical and Applied Genetics*.

[382] Arbelbide M, Bernardo R (2006). Mixed-model QTL mapping for kernel hardness and dough strength in bread wheat. *Theoretical and Applied Genetics*.

[383] Dobrovolskaya O, Arbuzova VS, Lohwasser U, Röder MS, Börner A (2006). Microsatellite mapping of complementary genes for purple grain colour in bread wheat (*Triticum aestivum*) L.. *Euphytica*.

[384] Nelson JC, Andreescu C, Breseghello F (2006). Quantitative trait locus analysis of wheat quality traits. *Euphytica*.

[385] Chen F, Luo Z, Zhang Z, Xia G, Min H (2007). Variation and potential value in wheat breeding of low-molecular-weight glutenin subunit genes cloned by genomic and RT-PCR in a derivative of somatic introgression between common wheat and *Agropyron elongatum*. *Molecular Breeding*.

[386] Pozniak CJ, Knox RE, Clarke FR, Clarke JM (2007). Identification of QTL and association of a phytoene synthase 
gene with endosperm colour in durum wheat. *Theoretical and Applied Genetics*.

[387] Cadalen T, Sourdille P, Charmet G (1998). Molecular markers linked to genes affecting plant height in wheat using a doubled-haploid population. *Theoretical and Applied Genetics*.

[388] Korzun V, Röder MS, Ganal MW, Worland AJ, Law CN (1998). Genetic analysis of the dwarfing gene (*Rht8*) in wheat—I: molecular mapping of *Rht8* on the short arm of chromosome 2D of bread wheat (*Triticum aestivum* L.). *Theoretical and Applied Genetics*.

[389] Ellis MH, Bonnett DG, Rebetzke GJ (2007). A 192bp allele at the Xgwm261 locus is not always associated with the *Rht8* dwarfing gene in wheat (*Triticum aestivum* L.). *Euphytica*.

[390] Kuraparthy V, Sood S, Dhaliwal HS, Chhuneja P, Gill BS (2007). Identification and mapping of a tiller inhibition gene (*t*
*i*
*n*3) in wheat. *Theoretical and Applied Genetics*.

[391] Kosuge K, Watanabe N, Kuboyama T (2008). Cytological and microsatellite mapping of mutant genes for spherical grain and compact spikes in durum wheat. *Euphytica*.

[392] Kato K, Miura H, Sawada S (1999). QTL mapping of genes controlling ear emergence time and plant height on chromosome 5A of wheat. *Theoretical and Applied Genetics*.

[393] Sourdille P, Snape JW, Cadalen T (2000). Detection of QTLs for heading time-and photoperiod response in wheat using a doubled-haploid population. *Genome*.

[394] Hanocq E, Niarquin M, Heumez E, Rousset M, Le Gouis J (2004). Detection and mapping of QTL for earliness components in a bread wheat recombinant inbred lines population. *Theoretical and Applied Genetics*.

[395] Xu X, Bai G, Carver BF, Shaner GE (2005). A QTL for early heading in wheat cultivar Suwon 92. *Euphytica*.

[396] Hanocq E, Laperche A, Jaminon O, Lainé A-L, Le Gouis J (2007). Most significant genome regions involved in the control of earliness traits in bread wheat, as revealed by QTL meta-analysis. *Theoretical and Applied Genetics*.

[397] Kato K, Miura H, Sawada S (2000). Mapping QTLs controlling grain yield and its components on chromosome 5A of wheat. *Theoretical and Applied Genetics*.

[398] Narasimhamoorthy B, Gill BS, Fritz AK, Nelson JC, Brown-Guedira GL (2006). Advanced backcross QTL analysis of a hard winter wheat × synthetic wheat population. *Theoretical and Applied Genetics*.

[399] Kirigwi FM, van Ginkel M, Brown-Guedira G, Gill BS, Paulsen GM, Fritz AK (2007). Markers associated with a QTL for grain yield in wheat under drought. *Molecular Breeding*.

[400] Kuchel H, Williams KJ, Langridge P, Eagles HA, Jefferies SP (2007). Genetic dissection of grain yield in bread wheat—I: QTL analysis. *Theoretical and Applied Genetics*.

[401] Ma Z, Zhao D, Zhang C (2007). Molecular genetic analysis of five spike-related traits in wheat using RIL and immortalized F_2_ populations. *Molecular Genetics and Genomics*.

[402] Kumar N, Kulwal PL, Gaur A (2006). QTL analysis for grain weight in common wheat. *Euphytica*.

[403] Kato K, Miura H, Akiyama M, Kuroshima M, Sawada S (1998). RFLP mapping of the three major genes, *Vrn1*, *Q* and *B1*, on the long arm of chromosome 5A of wheat. *Euphytica*.

[404] Peng ZS, Yen C, Yang JL (1998). Chromosomal location of genes for supernumerary spikelet in bread wheat. *Euphytica*.

[405] Salina E, Börner A, Leonova I (2000). Microsatellite mapping of the induced sphaerococcoid mutation genes in *Triticum aestivum*. *Theoretical and Applied Genetics*.

[406] Bullrich L, Appendino ML, Tranquilli G, Lewis S, Dubcovsky J (2002). Mapping of a thermo-sensitive earliness per se gene on *Triticum monococcum* chromosome 1Am. *Theoretical and Applied Genetics*.

[407] Khlestkina EK, Pestsova EG, Röder MS, Börner A (2002). Molecular mapping, phenotypic expression and geographical distribution of genes determining anthocyanin pigmentation of coleoptiles in wheat (*Triticum aestivum* L.). *Theoretical and Applied Genetics*.

[408] Xing QH, Ru ZG, Zhou CJ (2003). Genetic analysis, molecular tagging and mapping of the thermo-sensitive genic male-sterile gene (*wtms1*) in wheat. *Theoretical and Applied Genetics*.

[409] Chu C-G, Faris JD, Friesen TL, Xu SS (2006). Molecular mapping of hybrid necrosis genes *Ne1* and *Ne2* in hexaploid wheat using microsatellite markers. *Theoretical and Applied Genetics*.

[410] Dobrovolskaya O, Pshenichnikova TA, Arbuzova VS, Lohwasser U, Röder MS, Börner A (2007). Molecular mapping of genes determining hairy leaf character in common wheat with respect to other species of the *Triticeae*. *Euphytica*.

[411] Houshmand S, Knox RE, Clarke FR, Clarke JM (2007). Microsatellite markers flanking a stem solidness gene on chromosome 3BL in durum wheat. *Molecular Breeding*.

[412] Keller M, Karutz Ch, Schmid JE (1999). Quantitative trait loci for lodging resistance in a segregating wheat × spelt population. *Theoretical and Applied Genetics*.

[413] Hai L, Guo H, Xiao S (2005). Quantitative trait loci (QTL) of stem strength and related traits in a doubled-haploid population of wheat (*Triticum aestivum* L.). *Euphytica*.

[414] Nalam VJ, Vales MI, Watson CJW, Kianian SF, Riera-Lizarazu O (2006). Map-based analysis of genes affecting the brittle rachis character in tetraploid wheat (*Triticum turgidum* L.). *Theoretical and Applied Genetics*.

[415] Rebetzke GJ, Ellis MH, Bonnett DG, Richards RA (2007). Molecular mapping of genes for coleoptile growth in bread wheat (*Triticum aestivum* L.). *Theoretical and Applied Genetics*.

[416] Zhang G, Mergoum M Molecular mapping of kernel shattering and its association with *Fusarium* head blight resistance in a Sumai3 derived population.

[417] Kato K, Kidou S, Miura H, Sawada S (2002). Molecular cloning of the wheat *CK2*
*α* gene and detection of its linkage with *Vrn-A1* on chromosome 5A. *Theoretical and Applied Genetics*.

[418] Liu Q, Ni Z, Peng H, Song W, Liu Z, Sun Q (2007). Molecular mapping of a dominant non-glaucousness gene from synthetic hexaploid wheat 
(*Triticum aestivum* L.): molecular mapping of non-glaucousness gene in wheat. *Euphytica*.

[419] Carrera A, Echenique V, Zhang W (2007). A deletion at the *Lpx*-*B1* locus is associated with low lipoxygenase activity and improved pasta color in durum wheat (*Triticum turgidum ssp. durum*). *Journal of Cereal Science*.

[420] He XY, He ZH, Zhang LP (2007). Allelic variation of *polyphenol oxidase (PPO)* genes located on chromosomes 2A and 2D and development of functional markers for the *PPO* genes in common wheat. *Theoretical and Applied Genetics*.

[421] Nakamura S, Komatsuda T, Miura H (2007). Mapping diploid wheat homologues of *Arabidopsis* seed ABA signaling genes and QTLs for seed dormancy. *Theoretical and Applied Genetics*.

[422] Raman R, Raman H, Martin P (2007). Functional gene markers for polyphenol oxidase locus in bread wheat (*Triticum aestivum* L.). *Molecular Breeding*.

[423] Yang D-L, Jing R-L, Chang X-P, Li W (2007). Identification of quantitative trait loci and environmental interactions for accumulation and remobilization of water-soluble carbohydrates in wheat (*Triticum aestivum* L.) stems. *Genetics*.

[424] Mohammedler V, Lukman R, Ortiz-Islas S (2004). Genetic and physical mapping of photoperiod insensitive gene *Ppd-B1* in common wheat. *Euphytica*.

[425] Raman H, Raman R, Wood R, Martin P (2006). Repetitive indel markers within the *ALMT1* gene conditioning aluminium tolerance in wheat 
(*Triticum aestivum* L.). *Molecular Breeding*.

[426] Zhou L-L, Bai G-H, Ma H-X, Carver BF (2007). Quantitative trait loci for aluminum resistance in wheat. *Molecular Breeding*.

[427] Jefferies SP, Pallotta MA, Paull JG (2000). Mapping and validation of chromosome regions conferring boron toxicity tolerance in wheat (*Triticum aestivum*). *Theoretical and Applied Genetics*.

[428] Tóth B, Galiba G, Fehér E, Sutka J, Snape JW (2003). Mapping genes affecting flowering time and frost resistance on chromosome 5B of wheat. *Theoretical and Applied Genetics*.

[429] Ma L, Zhou E, Huo N, Zhou R, Wang G, Jia J (2007). Genetic analysis of salt tolerance in a recombinant inbred population of wheat 
(*Triticum aestivum* L.). *Euphytica*.

[430] Peng J, Ronin Y, Fahima T (2003). Domestication quantitative trait loci in *Triticum dicoccoides*, the progenitor of wheat. *Proceedings of the National Academy of Sciences of the United States of America*.

[431] Pozzi C, Rossini L, Vecchietti A, Salamini F, Gupta PK, Varshney RK (2004). Gene and genome changes during domestication of cereals. *Cereal Genomics*.

[432] Dubcovsky J, Dvorak J (2007). Genome plasticity a key factor in the success of polyploid wheat under domestication. *Science*.

[433] Uauy C, Brevis JC, Dubcovsky J (2006). The high grain protein content gene Gpc-B1 accelerates senescence and has pleiotropic effects on protein content in wheat. *Journal of Experimental Botany*.

[434] Flint-Garcia SA, Thuillet A-C, Yu J (2005). Maize association population: a high-resolution platform for quantitative trait locus dissection. *The Plant Journal*.

[435] Yu J, Buckler ES (2006). Genetic association mapping and genome organization of maize. *Current Opinion in Biotechnology*.

[436] Breseghello F, Sorrells ME (2006). Association mapping of kernel size and milling quality in wheat (*Triticum aestivum* L.) cultivars. *Genetics*.

[437] Ravel C, Praud S, Murigneux A (2006). Identification of *Glu-B1-1* as a candidate gene for the quantity of high-molecular-weight glutenin in bread wheat (*Triticum aestivum* L.) by means of an association study. *Theoretical and Applied Genetics*.

[438] Crossa J, Burgueño J, Dreisigacker S (2007). Association analysis of historical bread wheat germplasm using additive genetic covariance of relatives and population structure. *Genetics*.

[439] Tommasini L, Schnurbusch T, Fossati D, Mascher F, Keller B (2007). Association mapping of *Stagonospora nodorum* blotch resistance in modern European winter wheat varieties. *Theoretical and Applied Genetics*.

[440] Dubcovsky J (2004). Marker-assisted selection in public breeding programs: the wheat experience. *Crop Science*.

[441] Sorrells ME (2007). Application of new knowledge, technologies, and strategies to wheat improvement. *Euphytica*.

[442] Eagles HA, Bariana HS, Ogbonnaya FC (2001). Implementation of markers in Australian wheat breeding. *Australian Journal of Agricultural Research*.

[443] Ogbonnaya FC, Subrahmanyam NC, Moullet O (2001). Diagnostic DNA markers for cereal cyst nematode resistance in bread wheat. *Australian Journal of Agricultural Research*.

[444] Landjeva S, Korzun V, Börner A (2007). Molecular markers: actual and potential contributions to wheat genome characterization and breeding. *Euphytica*.

[445] Kuchel H, Ye G, Fox R, Jefferies S (2005). Genetic and economic analysis of a targeted marker-assisted wheat breeding strategy. *Molecular Breeding*.

[446] Kuchel H, Fox R, Reinheimer J (2007). The successful application of a marker-assisted wheat breeding strategy. *Molecular Breeding*.

[447] William HM, Trethowan R, Crosby-Galvan EM (2007). Wheat breeding assisted by markers: CIMMYT's experience. *Euphytica*.

[448] Lange C, Whittaker JC (2001). On prediction of genetic values in marker-assisted selection. *Genetics*.

[449] Radovanovic N, Cloutier S (2003). Gene-assisted selection for high molecular weight glutenin subunits in wheat doubled haploid breeding programs. *Molecular Breeding*.

[450] Bowman CM, Howe CJ, Dyer TA, Miller TE, Koebner RMD Molecular mechanisms contributing to the evolution of (wheat) chloroplast genomes.

[451] Ogihara Y, Tsunewaki K (2005). Genome science of polyploid wheat. *Frontiers of Wheat Bioscience. The 100th Memorial Issue of Wheat Information Service*.

[452] Tsunewaki K, Raupp WJ, Gill BS Plasmon differentiation in *Triticum* and *Aegilops* revealed by cytoplasmic effects on the wheat genome manifestation.

[453] Newton KJ (1988). Plant mitochondrial genomes: organization, expression and variation. *Annual Review of Plant Physiology and Plant Molecular Biology*.

